# Systems Analysis of a Mouse Xenograft Model Reveals Annexin A1 as a Regulator of Gene Expression in Tumor Stroma

**DOI:** 10.1371/journal.pone.0043551

**Published:** 2012-10-15

**Authors:** Ming Yi

**Affiliations:** Sidney Kimmel Cancer Center and Proteogenomics Research Institute for Systems Medicine, San Diego, California, United States of America; University Hospital Hamburg-Eppendorf, Germany

## Abstract

Annexin A1 is a multi functional molecule which is involved in inflammation, innate and adaptive immune systems, tumor progression and metastasis. We have previously showed the impaired tumor growth, metastasis, angiogenesis and wound healing in annexin A1 knockout mice. While tumor is a piece of heterogeneous mass including not only malignant tumor cells but also the stroma, the importance of the tumor stroma for tumor progression and metastasis is becoming increasingly clear. The tumor stroma is comprised by various components including extracellular matrix and non-malignant cells in the tumor, such as endothelial cells, fibroblasts, immune cells, inflammatory cells. Based on our previous finding of pro-angiogenic functions for annexin A1 in vascular endothelial cell sprouting, wound healing, tumor growth and metastasis, and the previously known properties for annexin A1 in immune cells and inflammation, this study hypothesized that annexin A1 is a key functional player in tumor development, linking the various components in tumor stroma by its actions in endothelial cells and immune cells. Using systems analysis programs commercially available, this paper further compared the gene expression between tumors from annexin A1 wild type mice and annexin A1 knockout mice and found a list of genes that significantly changed in the tumor stroma that lacked annexin A1. This revealed annexin A1 to be an effective regulator in tumor stroma and suggested a mechanism that annexin A1 affects tumor development and metastasis through interaction with the various components in the microenvironment surrounding the tumor cells.

## Introduction

We recently showed the pro-angiogenic functions in tumor development for annexin A1 [1] which was previously known as an inflammatory protein. Tumor growth and metastasis were significantly decreased when tumors grew in host animals unable to express annexin A1 [1]. While tumor is a piece of heterogeneous mass including not only malignant tumor cells but also the stroma, the importance of the tumor stroma for tumor progression and metastasis is becoming increasingly clear. The tumor stroma is comprised by extracellular matrix and non-malignant cells in the tumor, which include endothelial cells, fibroblasts, immune cells, inflammatory cells such as macrophages [2], [3]. Although the tumor stroma has been sought after as a therapeutic target, the mechanisms for how the interaction of various components in the complex tumor stroma contributes to the tumor development are still poorly understood. Based on our recent finding of pro-angiogenic functions for annexin A1 in vascular endothelial cell sprouting, wound healing, and tumor growth and metastasis [1], and the previously known properties for annexin A1 in immune cells and inflammation [4], this study hypothesized that annexin A1 is a key functional player in tumor development, linking the various components in tumor stroma by its actions in endothelial cells and immune cells. Here, we used systems biology approach to analyze the tumors in whole animal models on the background of absence or presence of annexin A1 to show the global effects of annexin A1 on tumor stroma. It is impossible to study tumor stroma with isolated proteins, cultured cell homogenates or even whole live cells. Tumor cells in culture lack the microenvironment or the stroma provided by the body that hosts the tumor. Our whole animal models offered significant advantages over any other conditions. Of particular importance our systems based approaches provided thorough analysis and generated interesting and thought-provoking results.

## Methods

### Mice and Tumor Models

Annexin A1 knockout homozygous and congenic wildtype counterpart homozygous mice, tumor cell culture, B16 melanoma cell line and subcutaneous tumor model were as described previously [1]. Briefly, to grow the xenograft tumors on the mice, B16 melanoma cells were grown in continuous culture for no more than 3 consecutive passages, the actively growing cells were then trypsinized (0.25% trypsin/1 mM Na-EDTA; Gibco/BRL), resuspended in DMEM (Dulbecco modified Eagle medium), counted, and injected subcutaneously into the right flanks of the mice at 5×10^6^ cells in 200 µL DMEM for each mouse. The B16 cell line was an established cell line and was obtained and used in Dr. Schnitzer's lab in Sidney Kimmel Cancer Center and Proteogenomics Research Institute for Systems Medicine, San Diego, California, United States of America, and the B16 cell line related work was previously published [1].

### Systems Analysis and Gene Microarray

The GeneChip mouse genome 430 2.0 array (Affymetrix) was used to analyze tumors from annexin A1 knockout or wildtype mice as described previously [1]. The dataset containing genes that are differentially expressed in the tumors from annexin A1 knockout mice and wildtype mice was analyzed using Genomatix software (Genomatix Software) and Gene Ontology database [5] and IPA software (Ingenuity Systems).

Study Approval. This study was approved by Sidney Kimmel Cancer Center and Proteogenomics Research Institute for Systems Medicine, San Diego, California, United States of America.

## Results

**Figure 1:**
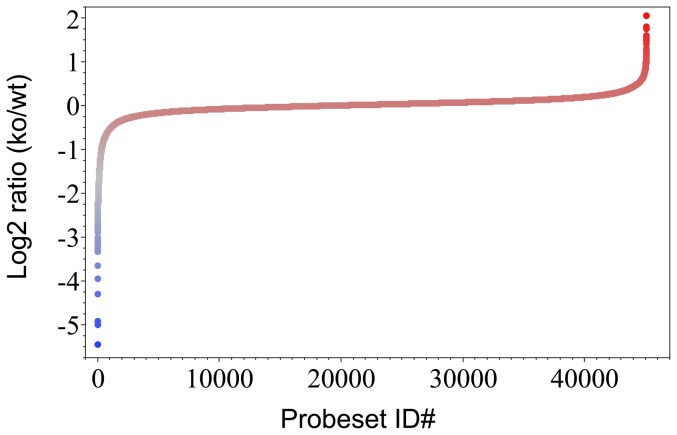
Gene differential expression profile of tumors from annexin A1 knockout and wild type mice. B16 melanoma cells were implanted in annexin A1 knockout and wild type mice to grow up tumors. Whole tumors were analyzed using mouse whole genome microarray, in which for each gene, one or more than one probeset(s) were spotted for the gene. For each probeset on the microarray, the difference in gene expression between tumors from annexin A1 knockout mice and from wild type mice was expressed as log 2 ratio of the gene expression level in annexin A1 knockout (ko) over the gene expression level in wild type (wt).

The tumors were grown in both annexin A1 null and wild type mice using B16 melanoma cells as described previously [1]. In this system, only the stroma in the tumor differs because the non-tumor cells are from the host body with or without annexin A1. From the raw data obtained previously [1] using mouse whole genome microarrays to run tumor samples from annexin A1 knockout and wildtype mice, this study performed thorough systems analysis. The gene expression levels in the whole tumors from annexin A1 knockout and wildtype mice were analyzed to obtain differential expression profiling (Fig. 1). For each gene probe in the mouse genome, the expression levels of the gene probe in tumors from annexin A1 null mice (ko) and from annexin A1 wild-type mice (wt) were compared and expressed as ratio of ko over wt, which reflects from three independent repeat experiments. In tumors from annexin A1 null mice, those genes with highest values were over-expressed (red colored region in Fig. 1) while those genes with lowest values were under-expressed (blue colored region in Fig. 1). The majority of the genome was expressed at similar extent in tumors from annexin A1 null and wild-type mice with the expression ratio values approaching one or the logarithm of the expression ratio values close to zero (Fig. 1). Total about 1000 genes with either highest or lowest ratio values were identified as significantly changed genes in the tumors.

**Figure 2:**
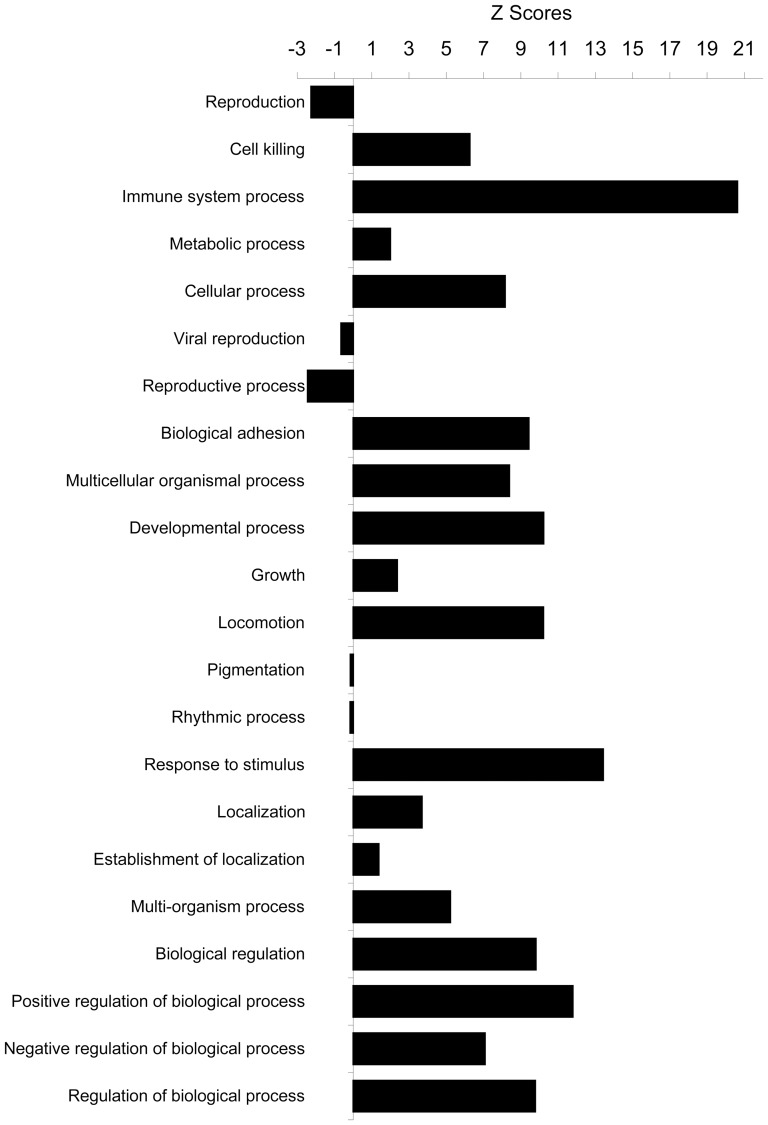
Biological process. The list of significantly changed genes was analyzed with Genomatix software and examined in terms of biological processes in Gene Ontology database and expressed as the Z score of a term for each biological process within the category. The higher value of Z score means more genes in that function of biological process were affected in this experiment, thus this biological process was over-represented. The Z scores for all the biological processes in top most general level of Gene Ontology structural networks of hierarchial tree of categories of biological process were shown here.

**Figure 3:**
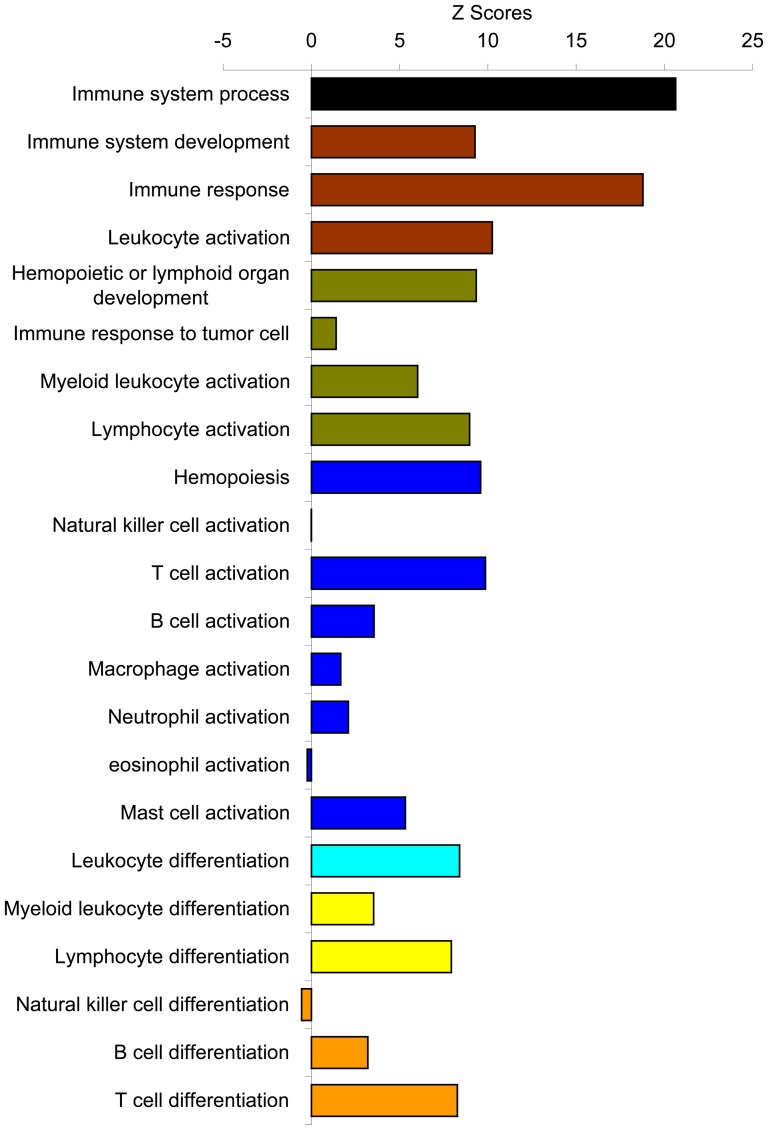
Immune system process. Mining down in Gene Ontology structural networks of hierarchial tree of categories of biological process, from top most general biological processes in Figure 2, the most over-represented immune system process was further mining down levels by levels into its subcategories with all biological processes in each level shown in Figure S1 and from each level, the over-represented biological processes are summarized here. Each color represents each level in the order from top to bottom as mining down the levels from top level of the hierarchial tree in the immune system process.

We then analyzed this list of significantly changed genes/proteins in a systems biology approach with Genomatix and IPA software to reveal the functional connection with annexin A1. The data were examined in terms of biological processes category, which are the Gene Ontology (GO) structured networks of defined terms to describe gene product attributes. The data were statistically analyzed and expressed as the z-score of a term to check for over- or under- represented groups of genes. Integrated statistical rating allows for the identification and selection of clusters of functionally related genes. All the biological processes in the GO database were obtained from top of the hierarchial tree to further mining down of subcategories of each branch levels by levels. The top most general biological processes are shown with their z-scores (Fig. 2). Interestingly, most of the biological processes were positively enriched, suggesting that annexin A1 may act as a global regulator which affects many biological processes and some of these processes may balance each other to result in minimum changes, which could contribute to the annexin A1 knockout mice appearing normal overall. The z-score of a term shows whether a certain gene, or group of genes, is over- or under-represented in the set of the genes being analyzed. Among the total 22 categories, the immune system process was most significantly over-represented (z-score, 20.63). This indicates that a group of genes, which are functionally related to immune system, were over-represented in the list of the differentially expressed genes identified above. This is consistent with the previously known functions of annexin A1 in immune system [4], thus the other genes related to immune system changed their expression levels in the tumors grown in the host microenvironment where annexin A1 was knocked out. Breakdown of this immune system process into its subcategories is shown in detail with the biological processes down levels by levels (Fig.S1) and shown in summary with representative biological processes (Fig. 3). The process of immune response to tumor cell was not much different between tumors in annexin A1 ko and wild-type conditions, consistent with our notion that it was the tumor stroma that made the tumor development significantly different in annexin A1 negative condition. Among the immune cells that were present in tumor stroma, T cell related processes stood out, which is consistent with the recently identified effects of annexin A1 on T cell functions [6].

**Figure 4:**
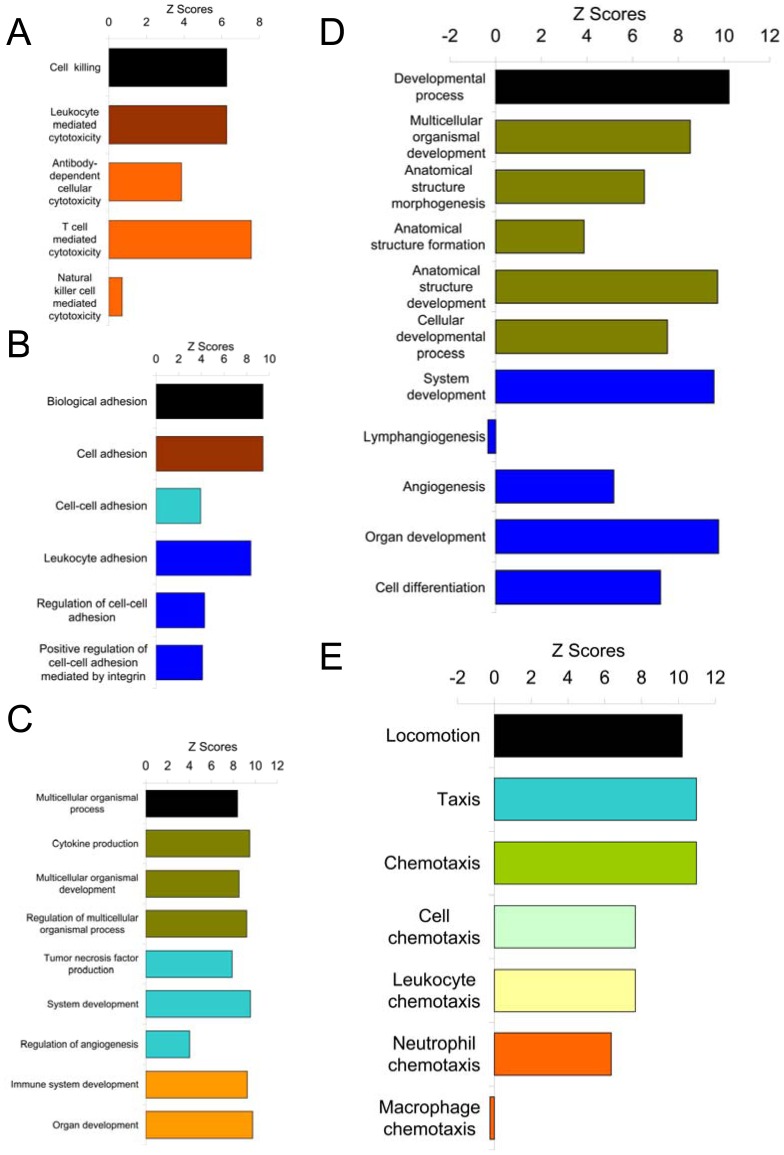
General biological processes other than immune system process. (A) Cell killing. (B) Biological adhesion. (C) Multicellular organismal process. (D) Developmental process. (E) Locomotion. Similarly as Figure 3, the most general processes were further mining down levels by levels into its subcategories with all biological processes in each level shown in Figures S2, S3, S4, S5, and S6 and from each level, the over-represented biological processes are summarized here. Each color represents each level in the order from top to bottom as mining down the levels from top level of the hierarchial tree in these biological processes.

**Figure 5:**
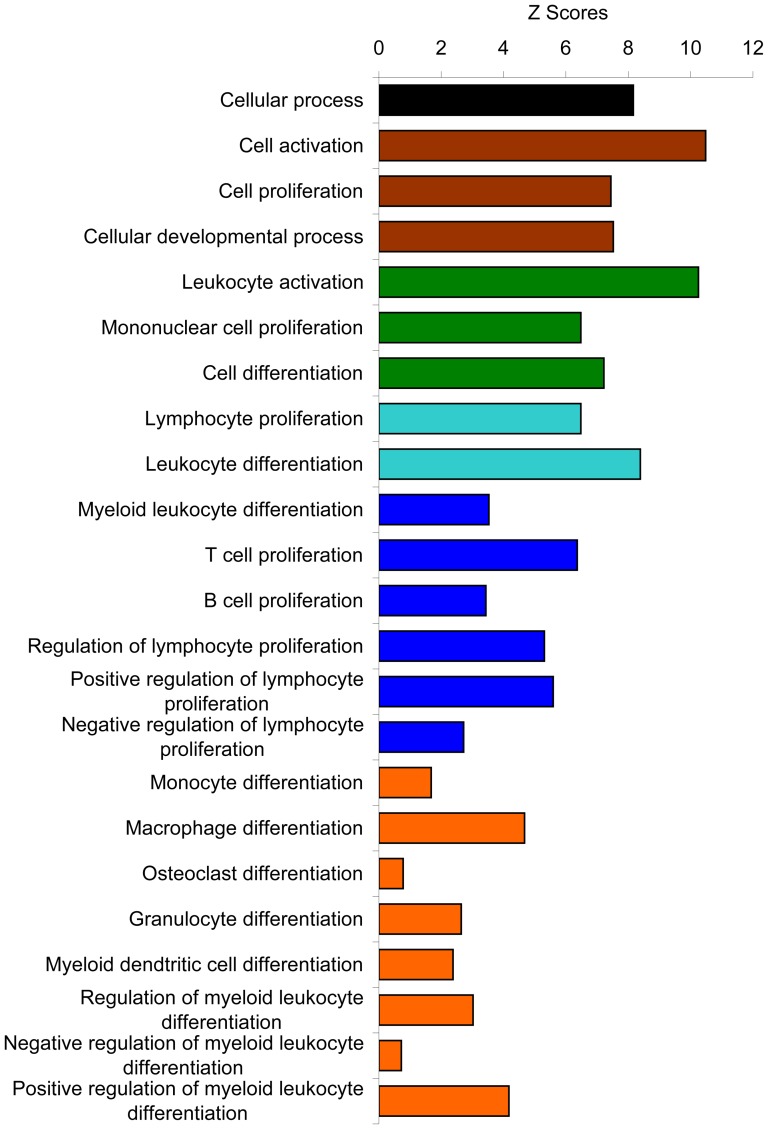
Cellular process. Similarly, from Figure 2, the cellular process was further mining down levels by levels into its subcategories with all biological processes in each level shown in Figure S7 and from each level, the over-represented biological processes are summarized here. Each color represents each level in the order from top to bottom as mining down the levels from top level of the hierarchial tree in the cellular process.

**Figure 6:**
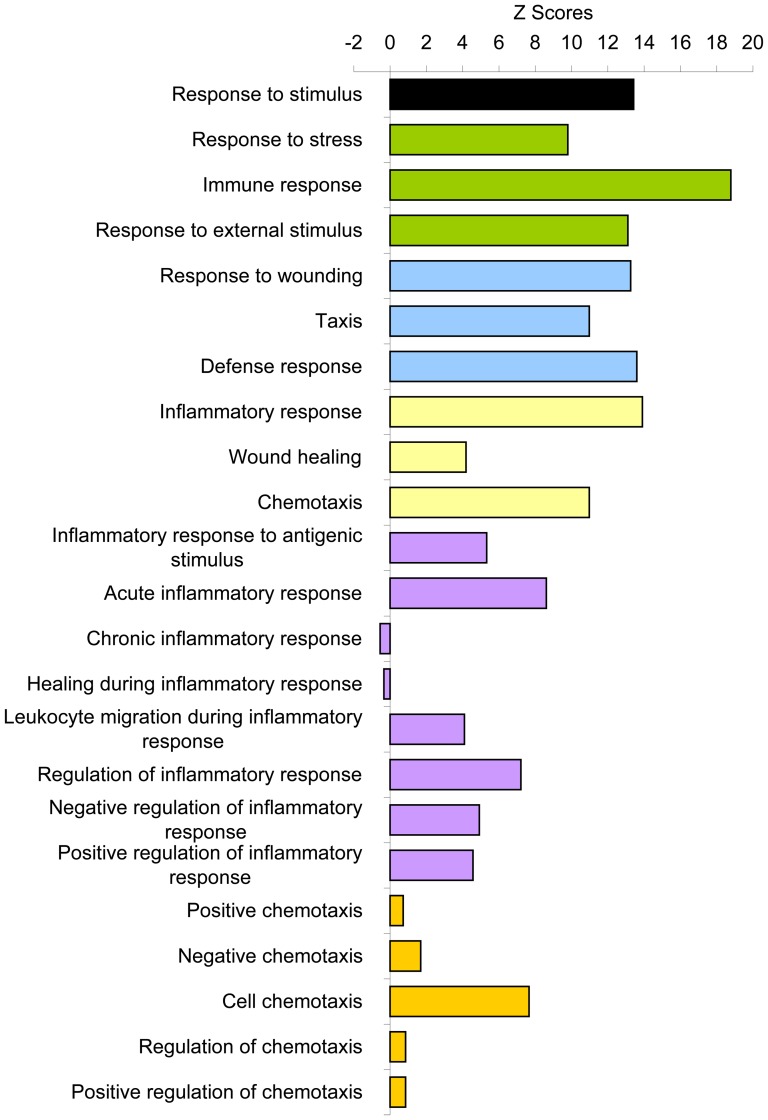
Response to stimulus. From Figure 2, the response to stimulus process was further mining down levels by levels into its subcategories with all biological processes in each level shown in Figure S8 and from each level, the over-represented biological processes are summarized here. Each color represents each level in the order from top to bottom as mining down the levels from top level of the hierarchial tree in the response to stimulus process.

**Figure 7:**
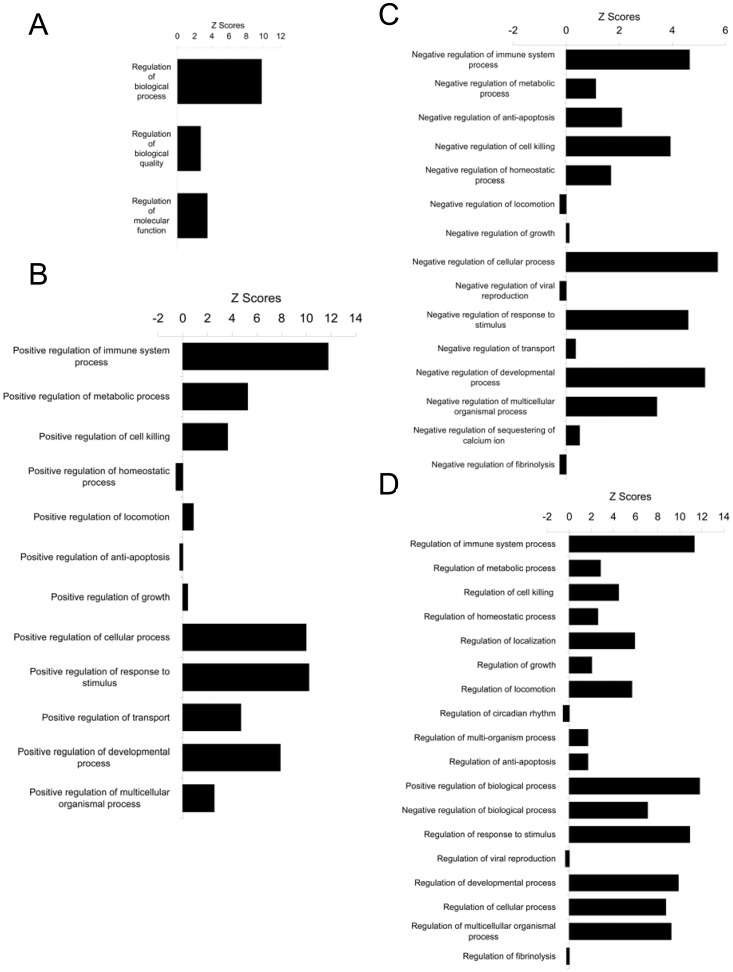
General biological processes other than immune system process. (A) Biological regulation. (B) Positive regulation of biological process. (C) Negative regulation of biological process. (D) Regulation of biological process. From Figure 2, these biological processes were further mining down into their subcategories.

Besides immune system process, other significantly over-represented general biological processes include cell killing, cellular process, biological adhesion, multicellular organismal process, developmental process, locomotion, response to stimulus, biological regulation, positive regulation of biological process, negative regulation of biological process, and regulation of biological process (Fig. 2). All of these processes were further mined down to their subcategories and the summary of the analyses were shown (Figs. 4, 5, 6, and 7) with the detailed analyses for each subcategory shown in supplementary information section (Figs.S2, S3, S4, S5, S6, S7, and S8). Mining down in the cell killing process (Fig. 4A), again, the category T cell mediated cytotoxicity was over-represented. Further highlighting the involvement of the immune, inflammatory components in the tumor stroma, the cellular process brought out the enriched process of macrophage differentiation (Fig. 5). Biological adhesion process (Fig. 4B) showed an over-representation of leukocyte adhesion, in line with the interaction between extracellular matrix and infiltrated non-tumor cells in tumor stroma. The process of tumor necrosis factor production was over-represented (Fig. 4C), consistent with our previous findings of increased necrosis in tumors grown in annexin A1 null mice [1], the change in tumor necrosis factor production may contribute to the amount of necrosis in tumors. As shown previously [1], angiogenesis was over-represented (Fig. 4D), the process involving endothelial cells in tumor stroma. The locomotion process (Fig. 4E) indicated an over-representation of leukocyte chemotaxis and neutrophil chemotaxis, both processes involving immune cells in tumor stroma. Again in the response to stimulus process (Fig. 6), the categories related to inflammatory response were significantly over-represented. It is noteworthy that the acute inflammatory response was also over-represented but not the chronic inflammatory response. Finally, biological processes related to regulation (Fig. 7), with overlapping subcategories showed a general over-representation of regulation for those over-represented biological processes discussed above. The positive regulation of biological process was more over-represented than the negative regulation of biological process.

**Figure 8:**
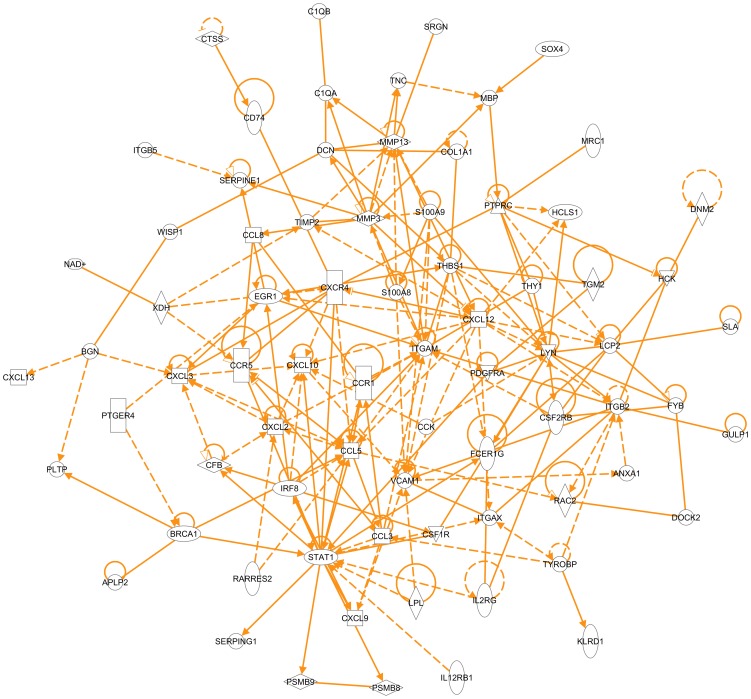
Tumor stroma interactome. The interaction network for most of the molecules on the list in Table 1 was built by IPA software (Ingenuity company).

**Figure 9:**
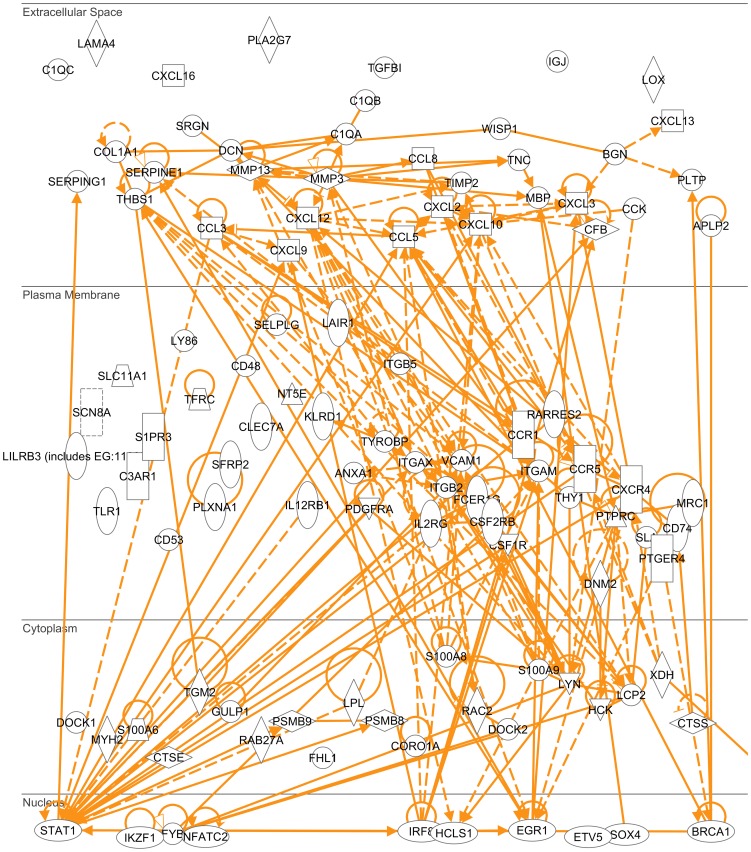
Tumor stroma interactome sub-cellular location. The sub-cellular distribution of the molecules on the interaction network (Figure 8) was built by IPA software (Ingenuity company).

Furthermore, we identified the molecules that were responsible for those over-represented biological processes to reveal an interactome in tumor stroma with annexin A1 being a key functional player. Using Genomatix and IPA software, we obtained lists of biological processes or functions with a list of molecules in each process or function. We combined the lists of molecules from the processes or functions that involve various components in tumor stroma to generate the list of molecules that comprises the tumor stroma interactome which interacts with annexin A1 (Table 1). These molecules showed most significant changes, either most down-regulated (log2 (ko/wt)<−0.999), or most up-regulated (log2 (ko/wt)>0.956), in the tumor stroma that lacked annexin A1. Based on the current literature data so far, most of the molecules on this list formed an interaction network built by IPA software (Fig. 8), of which, the interaction between annexin A1 and integrin beta 2 and the interaction between annexin A1 and vascular cell adhesion molecule 1 were identified based on current data. This interaction may occur in cell plasma membrane (Fig. 9). The expression ratio of integrin beta 2 in tumors from annexin A1 ko versus wt was 0.378 (Table 1), so the integrin beta 2 was down-regulated in the absence of annexin A1; and the vascular cell adhesion molecule 1 was even down-regulated (ratio of ko versus wt was 0.214).

**Table 1:** Tumor Stroma Interactome for Annexin A1.

Gene Symbol	Gene Name/Gene Title	Entrez Gene	ko/wt
Igh-6	immunoglobulin heavy chain 6 (heavy chain of IgM)	16019	0.065
Tgfbi	transforming growth factor, beta induced	21810	0.108
H2-Eb1	histocompatibility 2, class II antigen E beta	14969	0.124
Cd74	CD74 antigen (invariant polypeptide of major histocompatibility complex, class II antigen-associated)	16149	0.143
Ptprc	protein tyrosine phosphatase, receptor type, C	19264	0.143
Mmp13	matrix metallopeptidase 13	17386	0.147
Pla2g7	phospholipase A2, group VII (platelet-activating factor acetylhydrolase, plasma)	27226	0.164
H2-Aa	histocompatibility 2, class II antigen A, alpha	14960	0.169
Cxcl13	chemokine (C-X-C motif) ligand 13	55985	0.170
H2-Ab1	histocompatibility 2, class II antigen A, beta 1	14961	0.199
Fpr-rs2	formyl peptide receptor, related sequence 2	14289	0.203
Mmp3	matrix metallopeptidase 3	17392	0.206
Sfrp2	secreted frizzled-related protein 2	20319	0.206
Vcam1	vascular cell adhesion molecule 1	22329	0.214
Tgm2	transglutaminase 2, C polypeptide	21817	0.227
H2-Q7	histocompatibility 2, Q region locus 7	100044020	0.227
Ly6a	lymphocyte antigen 6 complex, locus A	110454	0.230
Ly86	lymphocyte antigen 86	17084	0.234
Igj	immunoglobulin joining chain	16069	0.237
Fcgr1	Fc receptor, IgG, high affinity I	14129	0.238
H2-DMa	histocompatibility 2, class II, locus DMa	14998	0.241
C1qb	complement component 1, q subcomponent, beta polypeptide	12260	0.244
C1qc	complement component 1, q subcomponent, C chain	12262	0.250
Hck	hemopoietic cell kinase	15162	0.256
Saa3	serum amyloid A 3	20210	0.257
Bcl2a1d	B-cell leukemia/lymphoma 2 related protein A1d	12047	0.258
Bcl2a1a	B-cell leukemia/lymphoma 2 related protein A1a	12044	0.258
Ccr5	chemokine (C-C motif) receptor 5	12774	0.261
Col1a1	procollagen, type I, alpha 1	12842	0.268
Cxcl16	chemokine (C-X-C motif) ligand 16	66102	0.271
Il2rg	interleukin 2 receptor, gamma chain	16186	0.281
C1qa	complement component 1, q subcomponent, alpha polypeptide	12259	0.288
Cxcl9	chemokine (C-X-C motif) ligand 9	17329	0.290
Ccl8	chemokine (C-C motif) ligand 8	100048554	0.290
Lpl	lipoprotein lipase	16956	0.291
Klra17	killer cell lectin-like receptor, subfamily A, member 17	170733	0.291
Egr1	early growth response 1	13653	0.296
Csf1r	colony stimulating factor 1 receptor	12978	0.297
Ctss	cathepsin S	13040	0.300
Itgax	integrin alpha X	16411	0.301
Coro1a	coronin, actin binding protein 1A	12721	0.301
Clec7a	C-type lectin domain family 7, member a	56644	0.301
Fcer1g	Fc receptor, IgE, high affinity I, gamma polypeptide	14127	0.308
Lcp2	lymphocyte cytosolic protein 2	16822	0.311
Tnc	tenascin C	21923	0.319
Irf8	interferon regulatory factor 8	15900	0.319
Srgn	serglycin	19073	0.322
Serpina3n	serine (or cysteine) peptidase inhibitor, clade A, member 3N	20716	0.325
Tyrobp	TYRO protein tyrosine kinase binding protein	22177	0.327
Ccl3	chemokine (C-C motif) ligand 3	20302	0.336
Cd53	CD53 antigen	12508	0.342
Cd48	CD48 antigen	12506	0.346
Fyb	FYN binding protein	23880	0.352
Ccl5	chemokine (C-C motif) ligand 5	20304	0.357
Fcgr3	Fc receptor, IgG, low affinity III	14131	0.365
Lox	lysyl oxidase	16948	0.365
Cxcl3	chemokinne (C-X-C motif) ligand 3	330122	0.369
C3ar1	complement component 3a receptor 1	12267	0.369
Slc11a1	solute carrier family 11 (proton-coupled divalent metal ion transporters), member 1	18173	0.374
Cxcr4	chemokine (C-X-C motif) receptor 4	12767	0.377
Thbs1	thrombospondin 1	640441	0.378
Itgb2	integrin beta 2	16414	0.378
Serping1	serine (or cysteine) peptidase inhibitor, clade G, member 1	12258	0.381
Cd24a	CD24a antigen	12484	0.382
Itgam	integrin alpha M	16409	0.386
Pdgfra	platelet derived growth factor receptor, alpha polypeptide	18595	0.392
Cxcl2	chemokine (C-X-C motif) ligand 2	20310	0.392
Il12rb1	interleukin 12 receptor, beta 1	16161	0.397
Hba-a2	hemoglobin alpha, adult chain 2	15122	0.407
Hba-a1	hemoglobin alpha, adult chain 1	110257	0.407
Serpine1	serine (or cysteine) peptidase inhibitor, clade E, member 1	18787	0.412
Ikzf1	IKAROS family zinc finger 1	22778	0.412
C3	complement component 3	12266	0.414
Dcn	decorin	13179	0.415
S100a6	S100 calcium binding protein A6 (calcyclin)	20200	0.429
Stat1	signal transducer and activator of transcription 1	20846	0.434
Psmb8	proteasome (prosome, macropain) subunit, beta type 8 (large multifunctional peptidase 7)	16913	0.441
Lair1	leukocyte-associated Ig-like receptor 1	52855	0.442
Cxcl4	chemokine (C-X-C motif) ligand 4	56744	0.443
Il2rg	interleukin 2 receptor, gamma chain	16186	0.444
Wisp1	WNT1 inducible signaling pathway protein 1	22402	0.444
Rarres2	retinoic acid receptor responder (tazarotene induced) 2	71660	0.448
S100a9	S100 calcium binding protein A9 (calgranulin B)	20202	0.451
Hcls1	hematopoietic cell specific Lyn substrate 1	15163	0.452
Cxcl10	chemokine (C-X-C motif) ligand 10	100045000	0.453
Lyn	Yamaguchi sarcoma viral (v-yes-1) oncogene homolog	676654	0.454
Ccr1	chemokine (C-C motif) receptor 1	12768	0.455
Klrd1	killer cell lectin-like receptor, subfamily D, member 1	16643	0.455
Csf2rb	colony stimulating factor 2 receptor, beta, low-affinity (granulocyte-macrophage)	12983	0.456
Selplg	selectin, platelet (p-selectin)ligand	20345	0.457
Pltp	phospholipid transfer protein	18830	0.457
Serpina1a	serine (or cysteine) peptidase inhibitor, clade A, member 1a	20700	0.459
S100a8	S100 calcium binding protein A8 (calgranulin A)	20201	0.460
Dock2	dedicator of cyto-kinesis 2	94176	0.462
Itgb5	integrin beta 5	16419	0.462
Thy1	thymus cell antigen 1, theta	21838	0.465
Tlr1	toll-like receptor 1	21897	0.466
H2-T23	histocompatibility 2, T region locus 23	100044190	0.474
Edg3	endothelial differentiation, sphingolipid G-protein-coupled receptor, 3	13610	0.476
Lilrb3	leukocyte immunoglobulin-like receptor, subfamily B (with TM and ITIM domains), member 3	100044531	0.478
Ctse	cathepsin E	13034	0.479
Xdh	xanthine dehydrogenase	22436	0.483
Etv5	ets variant gene 5	104156	0.484
Nt5Ee	5′ nucleotidase, ecto	23959	0.484
SaSh3	SAM and SH3 domain containing 3	74131	0.489
Ptger4	prostaglandin E receptor 4 (subtype EP4)	19219	0.491
Sla	src-like adaptor	20491	0.492
Bgn	biglycan	12111	0.494
Psmb9	proteasome (prosome, macropain) subunit, beta type 9 (large multifunctional peptidase 2)	16912	0.495
Cfb	complement factor B	14962	0.497
Rac2	RAS-related C3 botulinum substrate 2	19354	0.499
Mrc1	mannose receptor, C type 1	17533	0.500
Cxcl12	chemokine (C-X-C motif) ligand 12	20315	0.500
Dnm2	dynamin 2	13430	1.906
Brca1	breast cancer 1	12189	1.917
Dock1	dedicator of cyto-kinesis 1	330662	1.922
Sox4	SRY-box containing gene 4	20677	1.940
Myh2	myosin, heavy polypeptide 2, skeletal muscle, adult	17882	1.963
Nfatc2	nuclear factor of activated T-cells, cytoplasmic, calcineurin-dependent 2	18019	1.981
Mbp	myelin basic protein	17196	1.986
Scn8a	sodium channel, voltage-gated, type VIII, alpha	20273	2.019
Lama4	laminin, alpha 4	16775	2.021
Rab27a	RAB27A, member RAS oncogene family	11891	2.025
Plxna1	plexin A1	18844	2.031
Fhl1	four and a half LIM domains 1	14199	2.077
Aplp2	amyloid beta (A4) precursor-like protein 2	11804	2.113
Gulp1	GULP, engulfment adaptor PTB domain containing 1	70676	2.222
Timp2	tissue inhibitor of metalloproteinase 2	21858	2.224
Tfrc	transferrin receptor	22042	2.967
Cck	cholecystokinin	12424	3.347

While the tumor cells were originally same before being implanted into annexin A1 ko and wt mice, therefore, the tumor stroma in annexin A1 ko and wt mice exhibited significant differences in the gene expression profiles between the host body with and without annexin A1, although the genes in tumor cells may change their expression levels due to the interaction between tumor cells and the tumor stroma. Annexin A1 is abundant in neutrophils, monocytes, macrophages in wild type animal models [4]. In the annexin A1 null animal model, these non-tumor cells infiltrated into tumor stroma do not express annexin A1, this lack of annexin A1 protein function reflected as the gene expression level changes of several other proteins. This network of changes indicated these important genes/proteins (Table 1) that are interacting with annexin A1 directly or indirectly. The interaction of annexin A1 with these proteins was previously unknown.

## Discussion

Through systems biology analysis, we found this list of genes (Table 1) that significantly changed in the tumor stroma that lacked annexin A1. This showed a mechanism that annexin A1 affects tumor development and metastasis through interaction with the various components in the microenvironment surrounding the tumor cells. Literature has also showed that these genes are associated with tumor and/or tumor stroma: IGH-6 (ko/wt = 0.065) with thymic lymphoma and lymphoblastic leukemia [7]–[8], TGFBI (ko/wt = 0.108) with neuroblastoma, lung carcinoma, ovarian and prostate cancers, renal, gastrointestinal and brain tumors, colon cancer [9]–[14], PTPRC (ko/wt = 0.143) with human breast tumor stroma [15], MMP13 (ko/wt = 0.147) with skin tumor stroma [16], CCK (ko/wt = 3.347) with human pancreatic cancer [17], TFRC (ko/wt = 2.967) with colon cancer, human esophageal squamous cell carcinoma, human brain tumors, mouse mammary adenocarcinoma and rodent liver tumor [18]–[22], TIMP2 (ko/wt = 2.224) with human colorectal cancer, human pancreatic carcinoma, human renal cell carcinoma and human squamous cervical carcinoma [23]–[26], BRCA1 (ko/wt = 1.917) with human breast tumor stroma [27].

It is worth noting that TGFBI, as an extracellular matrix protein, has been reported differently either as a tumor suppressor [12], [28] or over-expressed in tumors [13]–[14]. TGFBI was shown to promote metastasis by acting on tumor stroma [14]. Here we showed that TGFBI was under-expressed in tumors from annexin A1 ko mice. In tumors grown on annexin A1 ko mice, the metastasis was reduced [1]. Thus, it suggests that annexin A1 interacts with or otherwise affects TGFBI and this interaction affects the metastatic ability of tumors. In addition, loss of TGFBI can induce resistance to chemotherapeutic agent paclitaxel in ovarian cancer cells [29], while human tumor cells with over-expressed levels of TGFBI showed an increased sensitivity to etoposide, paclitaxel, cisplatin and gemcitabine [10]. Similarly, stroma-derived annexin A1 has been shown to play a role in γ-irradiation-induced T-cell lymphoblastic lymphoma development, with annexin A1 being a candidate resistance gene against γ-radiation exposure [30]. It has been reviewed that the cellular and non-cellular components of the tumor microenvironment contribute to the chemoresistance [31]. Therefore, annexin A1 and TGFBI may be further studied together to elicit a mechanism for drug resistance. Annexin A1 and TGFBI could be important therapy targets to manipulate to improve the efficacy of radio-and chemo-therapies for cancer patients, to reduce resistance to treatments and decrease recurrence of cancer.

Protein tyrosine phosphatase gene expression was shown to be up-regulated in stromal fibroblasts from human breast tumors [15], while our study showed PTPRC down-regulated in tumor stroma without annexin A1 (Table 1), this suggests that inhibiting annexin A1 may disrupt tumor stroma to hamper tumor progression via decreasing expression level of protein tyrosine phosphatase. MMP13 was shown to promote angiogenesis [16], which is consistent with the finding in this study of down-regulated MMP13 expression (Table 1) and impaired tumor growth and angiogenesis in tumors on annexin A1 knockout mice [1].

The role of endogenous cholecystokinin (CCK) in human pancreatic cancer is not clear. The pancreatic cancer cells produced both CCK and gastrin, however the CCK level was lower than the gastrin. It seemed gastrin played a more dominant role than CCK in stimulating tumor growth. While down-regulation of gastrin inhibited growth of pancreatic tumor, change in the CCK level did not affect the tumor growth [17]. Here we showed a huge increase of CCK mRNA expression in tumors from annexin A1 ko mice, the further study of interaction of annexin A1 and CCK will shed light on understanding the role of CCK in human cancers.

Also, this study showed a possible interaction between annexin A1 and BRCA1. It has been shown that in the breast cancers containing mutated BRCA1, a common breast cancer susceptibility gene, changes in the tumor stroma facilitate the malignant transformation of the tumor cells [32]. BRCA1 is also believed to be a regulator in mammary stem cell differentiation and associated with cancer stem cells. One of the identified markers for selection of human stem cells and cancer stem cells is aldehyde dehydrogenase 1 (ALDH1), however, the exact function of ALDH1 in stem cells is still mostly unknown. ALDH1 expression is significantly increased in both tumor cells and tumor stroma in breast cancers carrying BRCA1 mutations [33]. The cancer stem cells possess tumor initiating capacity and therapy resistance. Therefore, consistent with the above mentioned annexin A1 being candidate resistance gene, an association between annexin A1 and stem cells and cancer stem cells is strongly possible and worth further investigation to help understand the exact functions of stem cell markers.

## Supporting Information

Figure S1 **Breakdown of immune system process category into its subcategories.** (A) Immune system process. (B1) Immune response. (B2) Leukocyte activation. (B3) Immune system development. (C1) Lymphocyte activation. (C2) Myeloid leukocyte activation. (C3) Regulation of leukocyte activation. (C4) Hemopoietic or lymphoid organ development. (D) Hemopoiesis. (E) Leukocyte differentiation. (F) Lymphocyte differentiation. Mining down in Gene Ontology structural networks of hierarchial tree of categories of biological process, the top level category, immune system process, labeled (A), was further mining down levels by levels into its subcategories with all biological processes in each category shown here, labeled alphabetically with each letter for each down level and for each level, representative categories were further broken down into all its subcategories shown here.
(PPT)

Figure S2 **Breakdown of cell killing category into its subcategories.** (A) Cell killing. (B) Leukocyte mediated cytotoxicity. (C) T cell mediated cytotoxicity. (D) Regulation of T cell mediated cytotoxicity. Similarly as Figure S1, the top level category, cell killing, labeled (A), was further mining down levels by levels into its subcategories labeled alphabetically with each letter for each down level and for each level, representative categories were further broken down into all its subcategories shown here.
(PPT)

Figure S3 **Breakdown of biological adhesion category into its subcategories.** (A) Biological adhesion. (B) Cell adhesion. (C1) Cell-cell adhesion. (C2) Cell-substrate adhesion. (C3) Regulation of cell adhesion. (C4) Positive regulation of cell adhesion. (C5) Negative regulation of cell adhesion. (D1) Leukocyte adhesion. (D2) Regulation of cell-substrate adhesion. (D3) Regulation of cell-cell adhesion. (D4) Regulation of cell adhesion mediated by integrin. (E1) Positive regulation of cell adhesion mediated by integrin. (E2) Regulation of cell-cell adhesion mediated by integrin. Similarly as Figure S1, the top level category, biological adhesion, labeled (A), was further mining down levels by levels into its subcategories labeled alphabetically with each letter for each down level and for each level, representative categories were further broken down into all its subcategories shown here.
(PPT)

Figure S4 **Breakdown of multicellular organismal process category into its subcategories.** (A) Multicellular organismal process. (B1) Cytokine production. (B2) System process. (B3) Multicellular organismal development. (B4) Tissue remodeling. (B5) Multicellular organismal homeostasis. (B6) Regulation of body fluid levels. (B7) Regulation of multicellular organismal process. (C1) Tumor necrosis factor production. (C2) System development. (C3) Negative regulation of tissue remodeling. (C4) Regulation of tissue remodeling. (C5) Regulation of angiogenesis. (D) Regulation of tumor necrosis factor production. (E) Regulation of tumor necrosis factor biosynthetic process. Similarly as Figure S1, the top level category, multicellular organismal process, labeled (A), was further mining down levels by levels into its subcategories labeled alphabetically with each letter for each down level and for each level, representative categories were further broken down into all its subcategories shown here.
(PPT)

Figure S5 **Breakdown of developmental process category into its subcategories.** (A) Developmental process. (B1) Multicellular organismal development. (B2) Anatomical structure morphogenesis. (B3) Anatomical structure formation. (B4) Anatomical structure development. (B5) Cellular developmental process. (B6) Regulation of developmental process. (B7) Negative regulation of developmental process. (B8) Positive regulation of developmental process. (C) Positive regulation of programmed cell death. (D1) Induction of programmed cell death. (D2) Positive Regulation of apoptosis. (E1) Induction of apoptosis. (E2) Positive regulation of lymphocyte apoptosis. (F1) Induction of apoptosis by extracellular signals. (F2) Induction of apoptosis by intracellular signals. Similarly as Figure S1, the top level category, developmental process, labeled (A), was further mining down levels by levels into its subcategories labeled alphabetically with each letter for each down level and for each level, representative categories were further broken down into all its subcategories shown here.
(PPT)

Figure S6 **Breakdown of locomotion category into its subcategories.** (A) Locomotion. (B1) Taxis. (B2) Regulation of locomotion. (B3) Cell motility. (C) Chemotaxis. (D) Cell chemotaxis. (E) Leukocyte chemotaxis. Similarly as Figure S1, the top level category, locomotion, labeled (A), was further mining down levels by levels into its subcategories labeled alphabetically with each letter for each down level and for each level, representative categories were further broken down into all its subcategories shown here.
(PPT)

Figure S7 **Breakdown of cellular process category into its subcategories.** (A) Cellular process. (B1) Cell activation. (B2) Cell motion. (B3) Cell communication. (B4) Cell adhesion. (B5) Cell proliferation. (B6) Cellular component organization. (B7) Cellular developmental process. (C1) Mononuclear cell proliferation. (C2) Cell differentiation. (D1) Lymphocyte proliferation. (D2) Leukocyte differentiation. (E) Myeloid leukocyte differentiation. Similarly as Figure S1, the top level category, cellular process, labeled (A), was further mining down levels by levels into its subcategories labeled alphabetically with each letter for each down level and for each level, representative categories were further broken down into all its subcategories shown here.
(PPT)

Figure S8 **Breakdown of response to stimulus category into its subcategories.** (A) Response to stimulus. (B1) Response to external stimulus. (B2) Response to stress. (C1) Defense response. (C2) Response to wounding. (C3) Taxis. (D1) Inflammatory response. (D2) Chemotaxis. (E) Acute inflammatory response. (F) Activation of plasma proteins during acute inflammatory response. (G) Complement activation. Similarly as Figure S1, the top level category, response to stimulus, labeled (A), was further mining down levels by levels into its subcategories labeled alphabetically with each letter for each down level and for each level, representative categories were further broken down into all its subcategories shown here.
(PPT)
