# Supplementary figures and images for: Systems Analysis of a Mouse Xenograft Model Reveals Annexin A1 as a Regulator of Gene Expression in Tumor Stroma
Source: PLoS One. 2012 Oct 15;7(10):e43551. doi: 10.1371/journal.pone.0043551 (PMC3471933; doi:10.1371/journal.pone.0043551)

## Slide 1
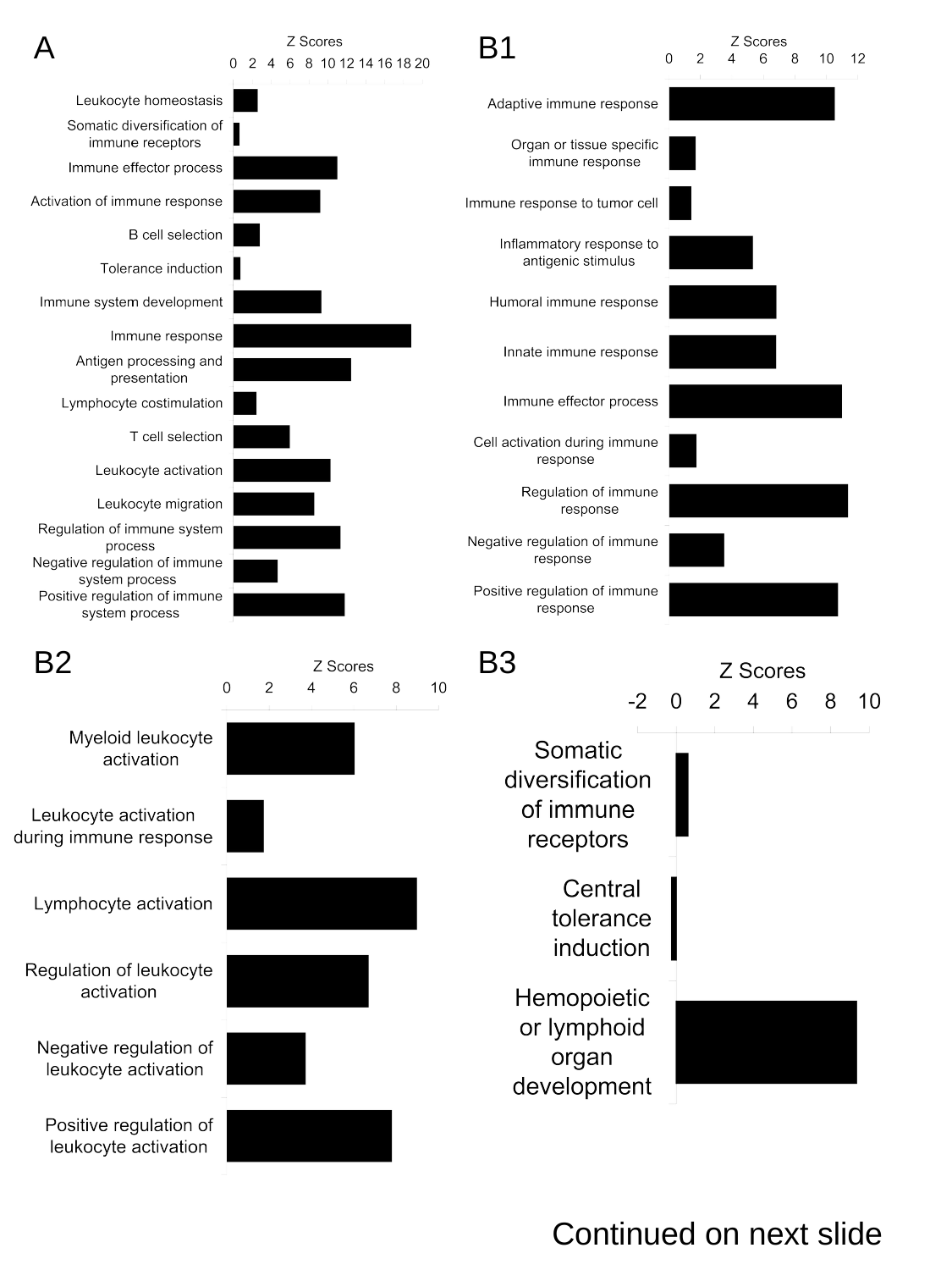

A
B1
B2
B3
Continued on next slide

## Slide 2
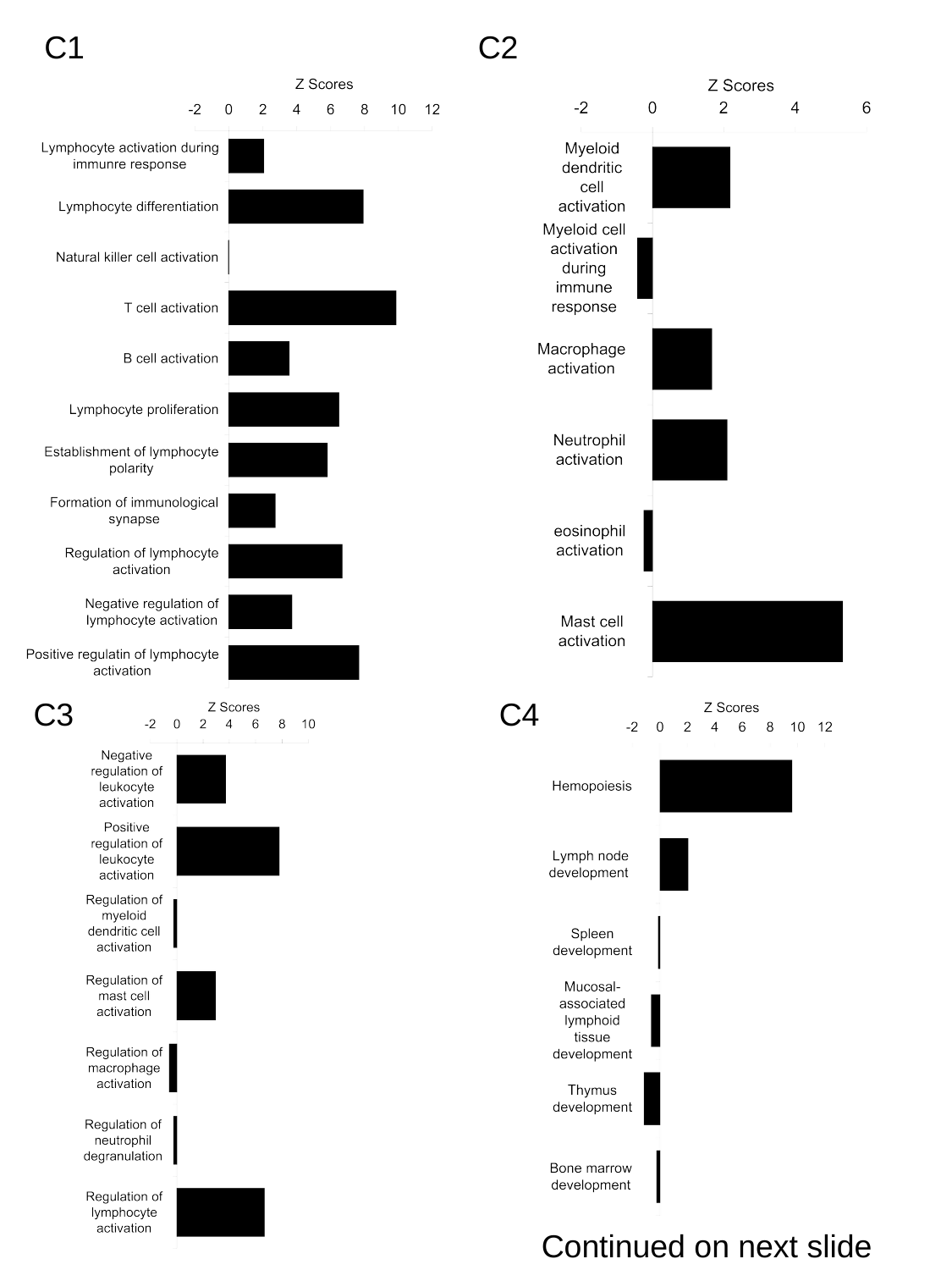

C1
C2
C3
C4
Continued on next slide

## Slide 3
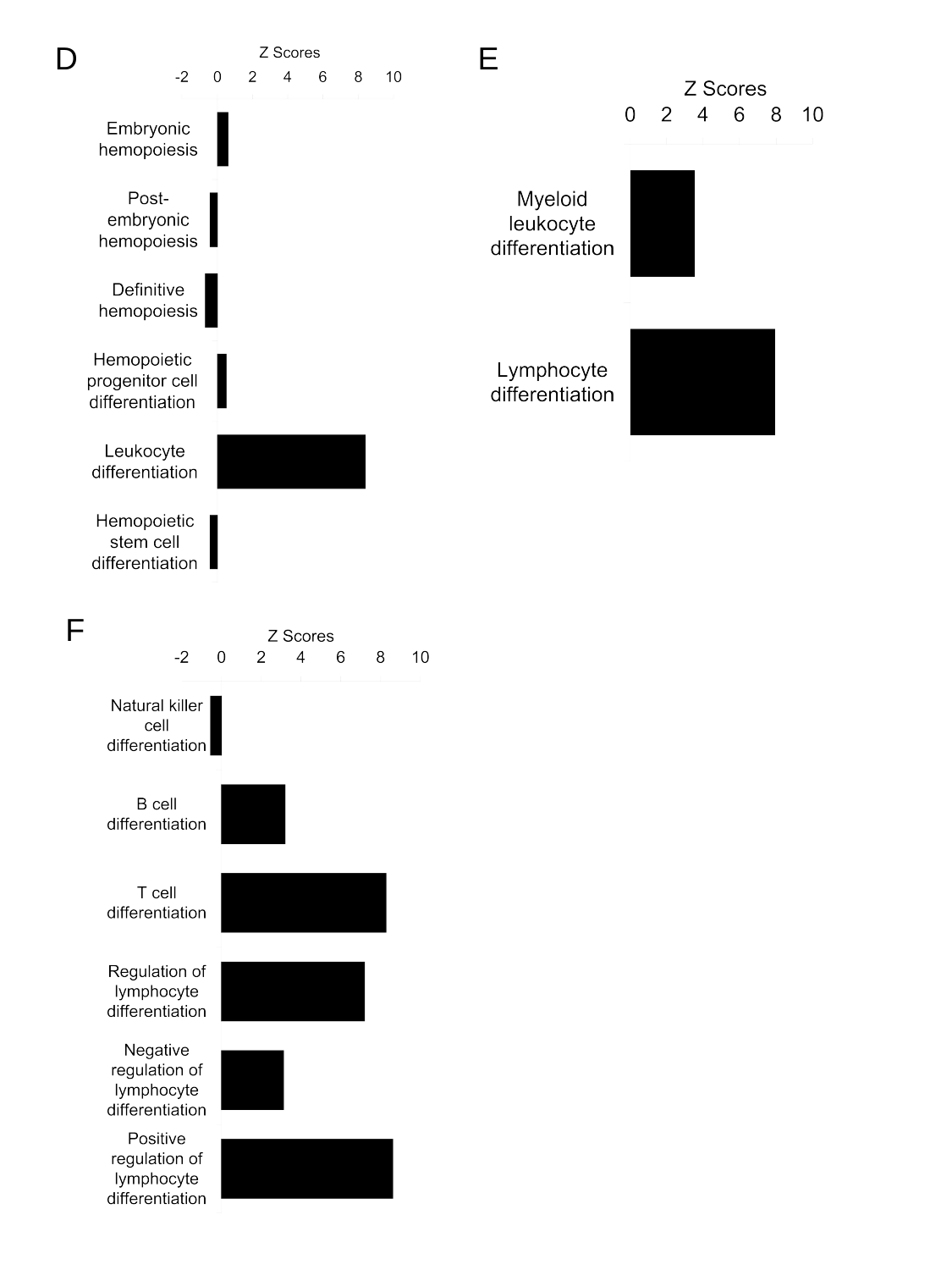

D
E
F

Supplement: Figure S1 — Breakdown of immune system process category into its subcategories. (A) Immune system process. (B1) Immune response. (B2) Leukocyte activation. (B3) Immune system development. (C1) Lymphocyte activation. (C2) Myeloid leukocyte activation. (C3) Regulation of leukocyte activation. (C4) Hemopoietic or lymphoid organ development. (D) Hemopoiesis. (E) Leukocyte differentiation. (F) Lymphocyte differentiation. Mining down in Gene Ontology structural networks of hierarchial tree of categories of biological process, the top level category, immune system process, labeled (A), was further mining down levels by levels into its subcategories with all biological processes in each category shown here, labeled alphabetically with each letter for each down level and for each level, representative categories were further broken down into all its subcategories shown here. (PPT) [file pone.0043551.s001.ppt]

## Slide 1
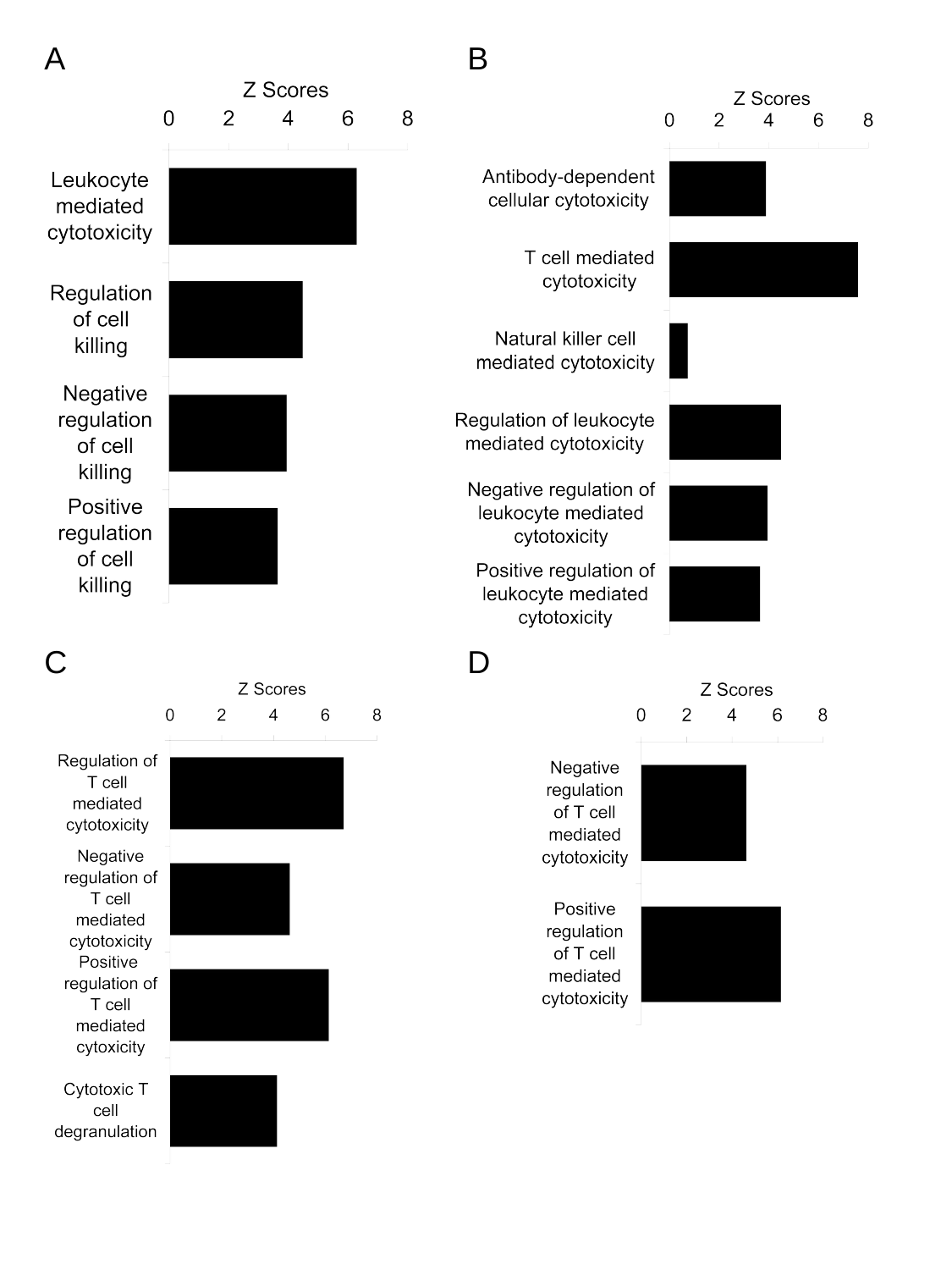

A
B
C
D

Supplement: Figure S2 — Breakdown of cell killing category into its subcategories. (A) Cell killing. (B) Leukocyte mediated cytotoxicity. (C) T cell mediated cytotoxicity. (D) Regulation of T cell mediated cytotoxicity. Similarly as Figure S1, the top level category, cell killing, labeled (A), was further mining down levels by levels into its subcategories labeled alphabetically with each letter for each down level and for each level, representative categories were further broken down into all its subcategories shown here. (PPT) [file pone.0043551.s002.ppt]

## Slide 1
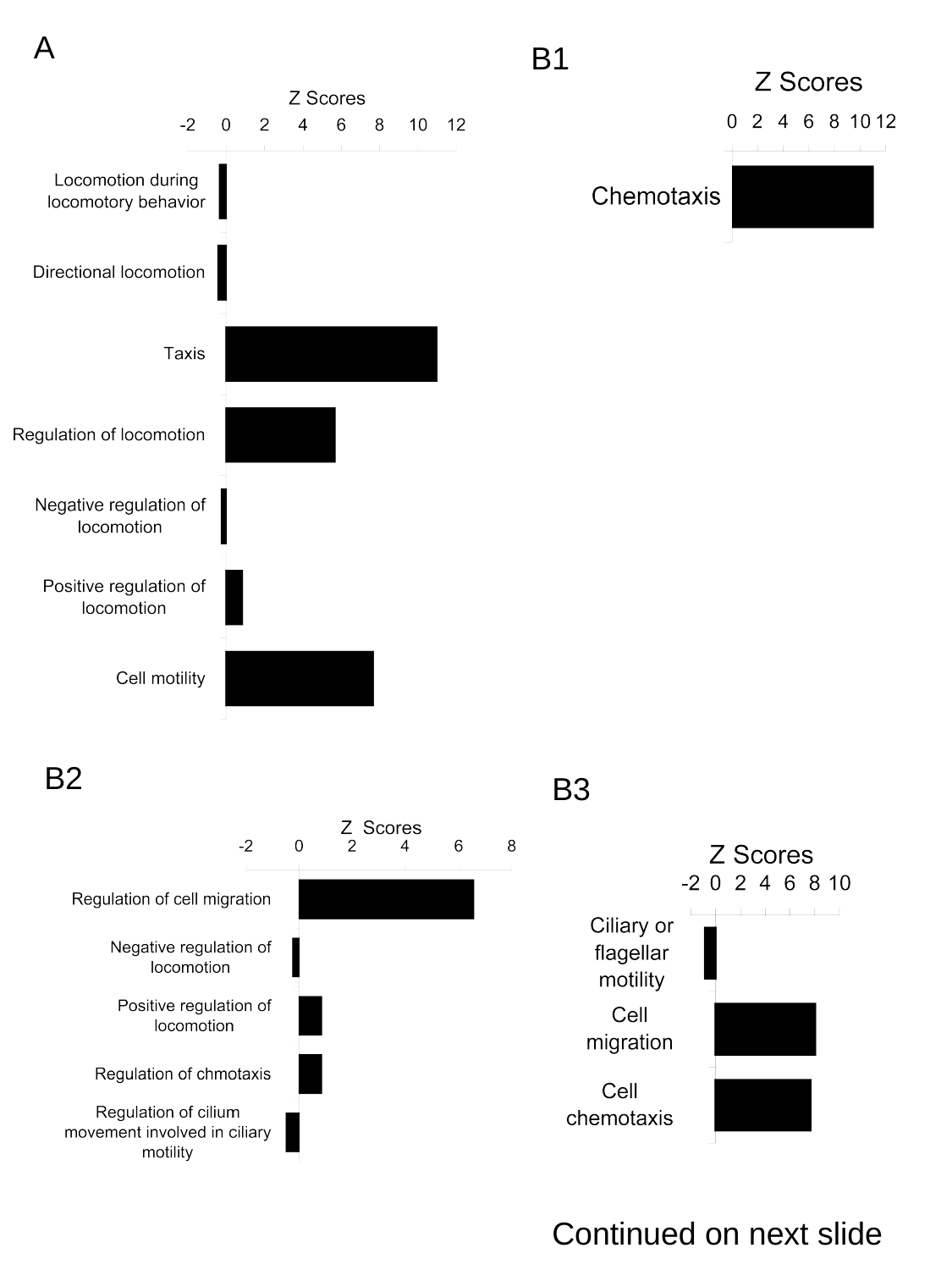

A
B1
B2
B3
Continued on next slide

## Slide 2
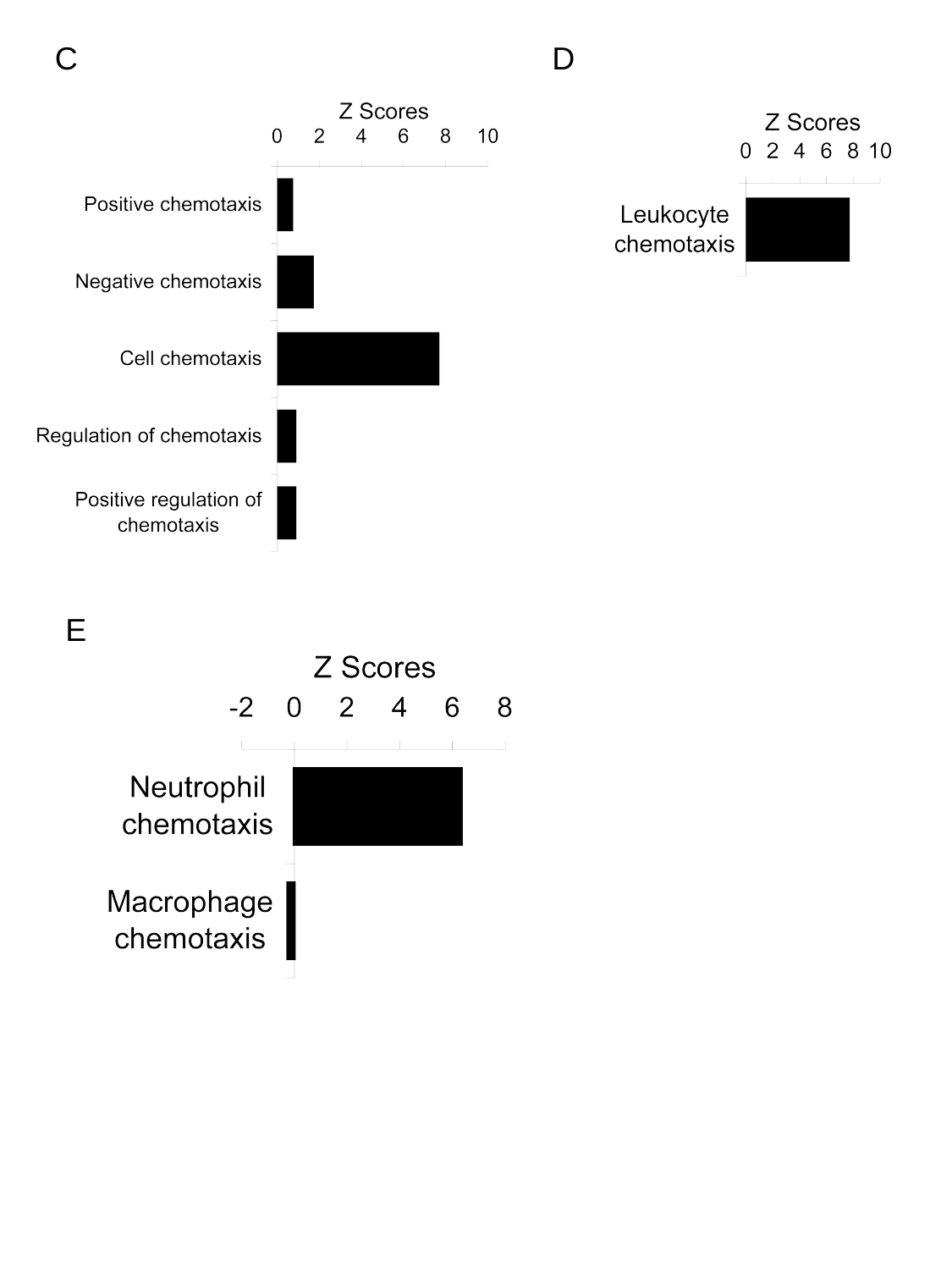

C
D
E

Supplement: Figure S6 — Breakdown of locomotion category into its subcategories. (A) Locomotion. (B1) Taxis. (B2) Regulation of locomotion. (B3) Cell motility. (C) Chemotaxis. (D) Cell chemotaxis. (E) Leukocyte chemotaxis. Similarly as Figure S1, the top level category, locomotion, labeled (A), was further mining down levels by levels into its subcategories labeled alphabetically with each letter for each down level and for each level, representative categories were further broken down into all its subcategories shown here. (PPT) [file pone.0043551.s006.ppt]
