# Supplementary material for: Systems Analysis of a Mouse Xenograft Model Reveals Annexin A1 as a Regulator of Gene Expression in Tumor Stroma
Source: PLoS One. 2012 Oct 15;7(10):e43551. doi: 10.1371/journal.pone.0043551 (PMC3471933; doi:10.1371/journal.pone.0043551)
Supplement: Figure S3 — Breakdown of biological adhesion category into its subcategories. (A) Biological adhesion. (B) Cell adhesion. (C1) Cell-cell adhesion. (C2) Cell-substrate adhesion. (C3) Regulation of cell adhesion. (C4) Positive regulation of cell adhesion. (C5) Negative regulation of cell adhesion. (D1) Leukocyte adhesion. (D2) Regulation of cell-substrate adhesion. (D3) Regulation of cell-cell adhesion. (D4) Regulation of cell adhesion mediated by integrin. (E1) Positive regulation of cell adhesion mediated by integrin. (E2) Regulation of cell-cell adhesion mediated by integrin. Similarly as Figure S1, the top level category, biological adhesion, labeled (A), was further mining down levels by levels into its subcategories labeled alphabetically with each letter for each down level and for each level, representative categories were further broken down into all its subcategories shown here. (PPT) [file pone.0043551.s003.ppt]

## Slide 1
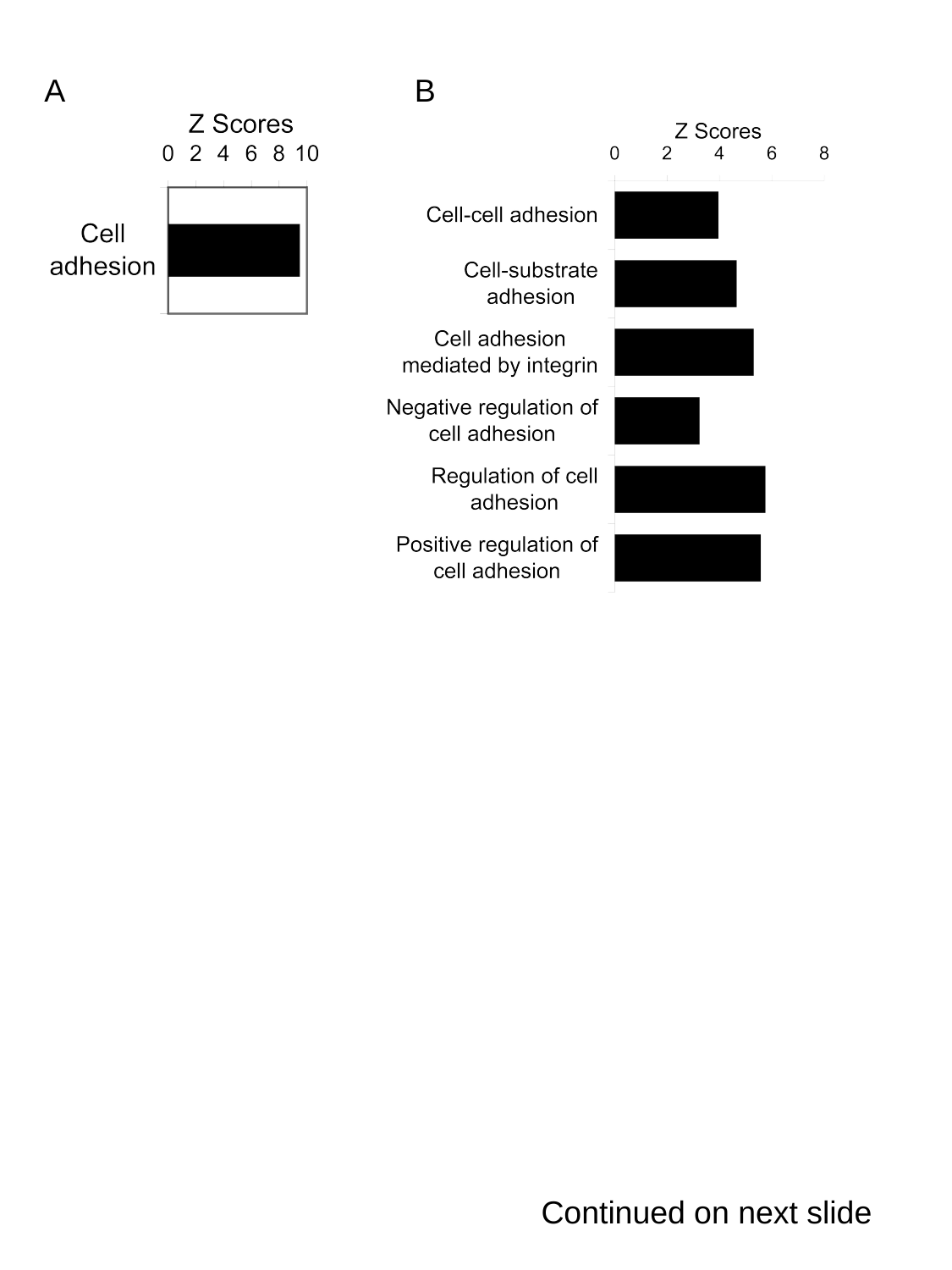

A
B
Continued on next slide

## Slide 2
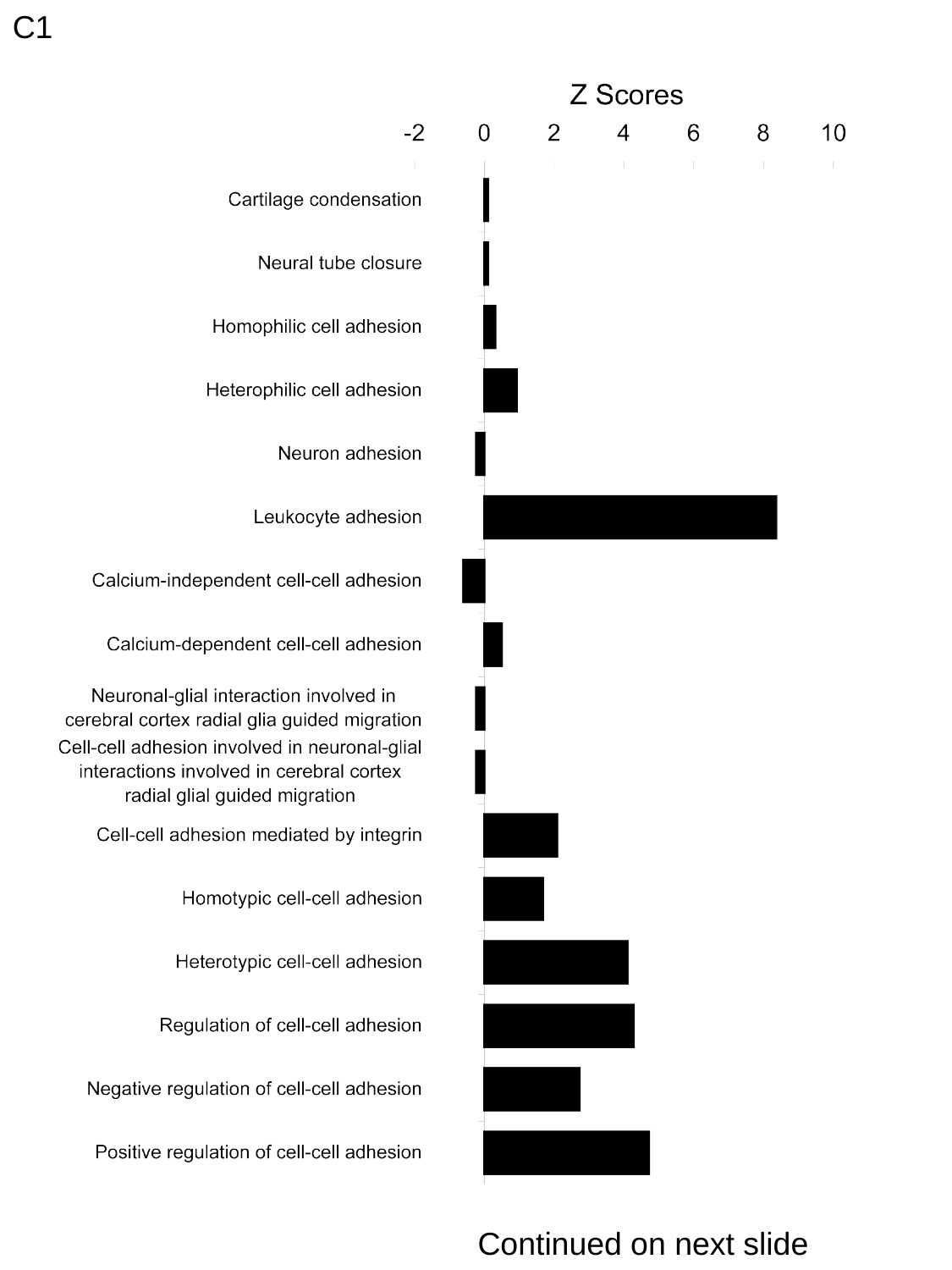

C1
Continued on next slide

## Slide 3
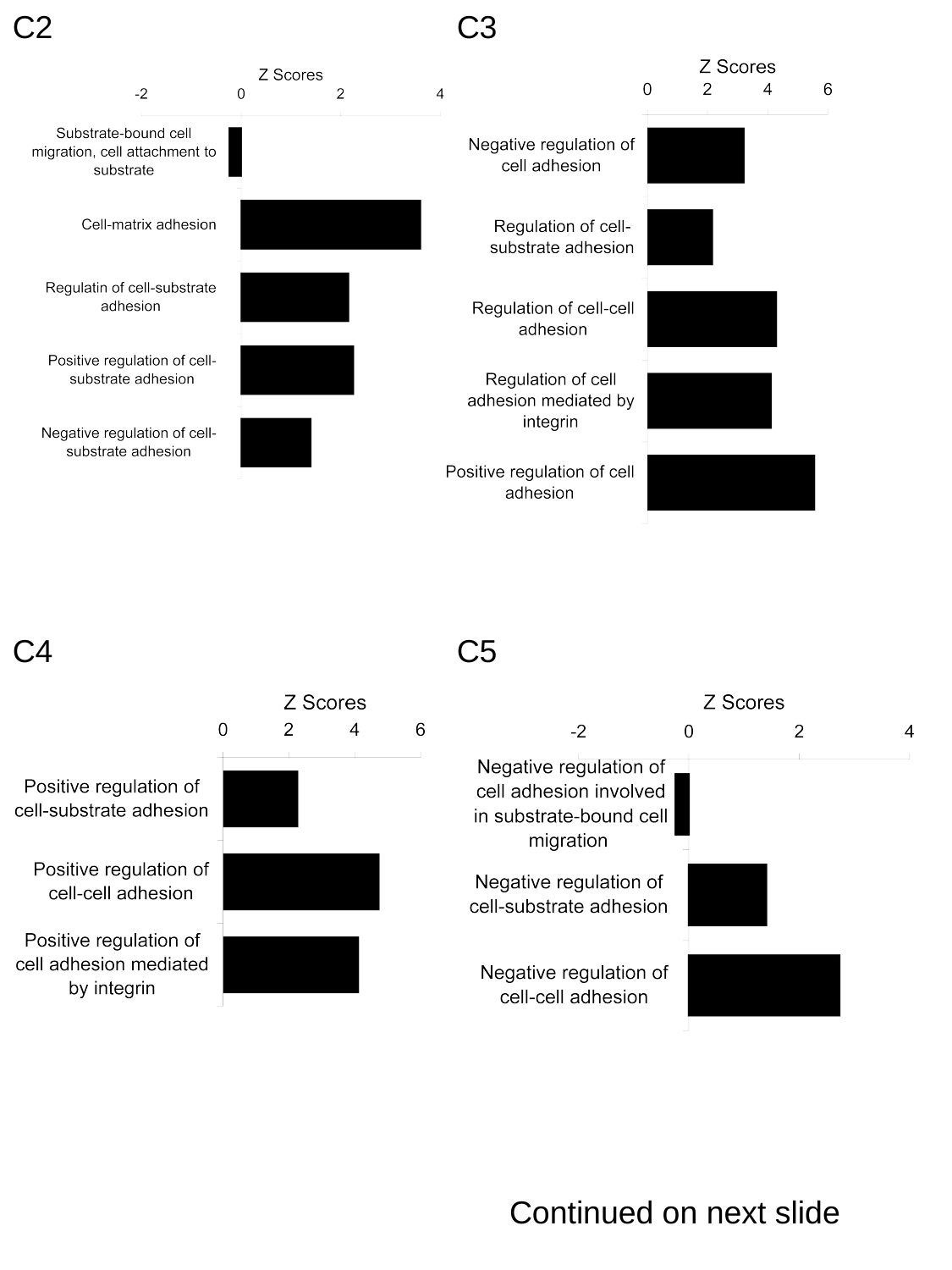

C2
C3
C4
C5
Continued on next slide

## Slide 4
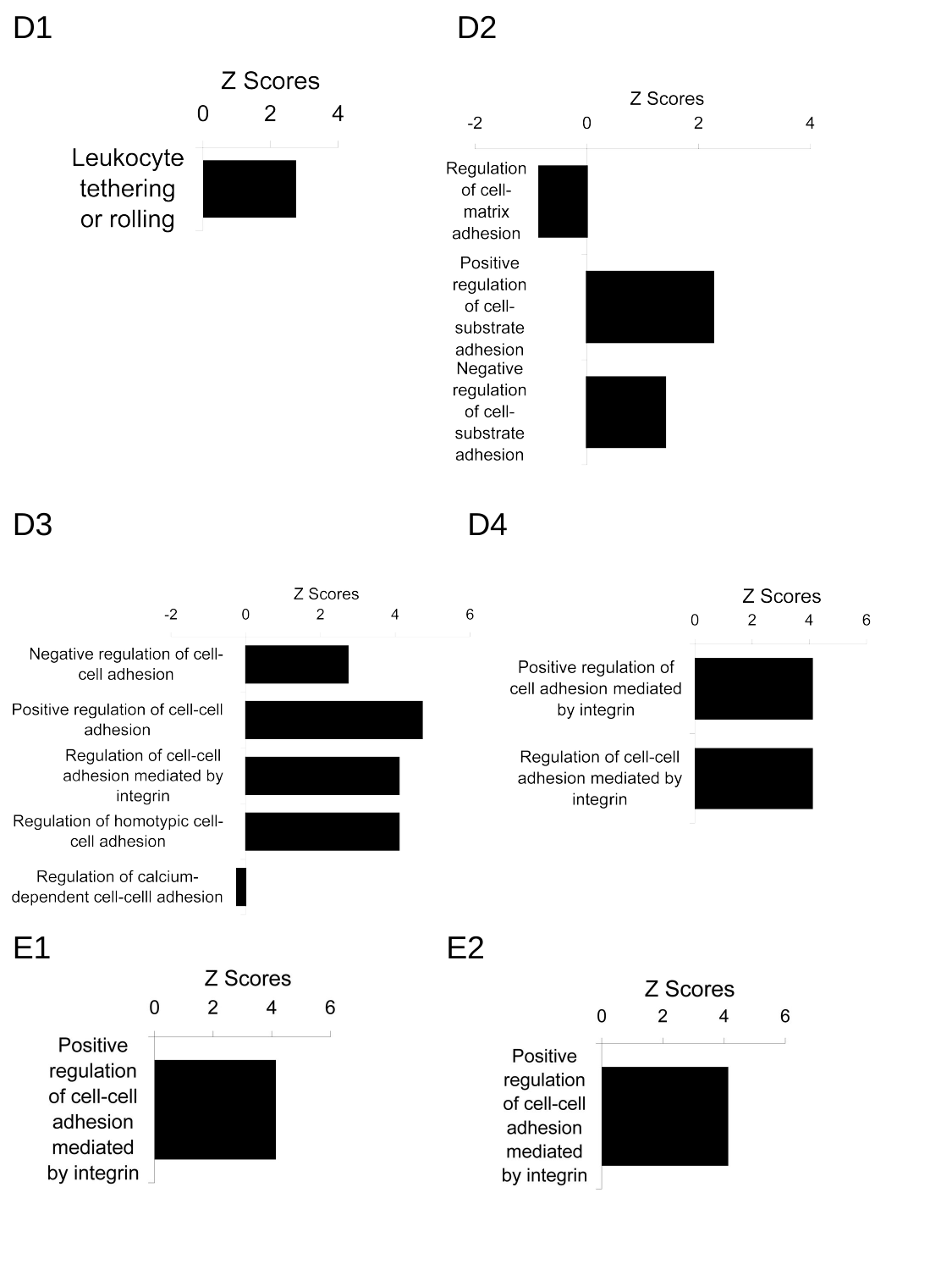

D1
D2
D3
D4
E1
E2
