# Supplementary material for: Systems Analysis of a Mouse Xenograft Model Reveals Annexin A1 as a Regulator of Gene Expression in Tumor Stroma
Source: PLoS One. 2012 Oct 15;7(10):e43551. doi: 10.1371/journal.pone.0043551 (PMC3471933; doi:10.1371/journal.pone.0043551)
Supplement: Figure S4 — Breakdown of multicellular organismal process category into its subcategories. (A) Multicellular organismal process. (B1) Cytokine production. (B2) System process. (B3) Multicellular organismal development. (B4) Tissue remodeling. (B5) Multicellular organismal homeostasis. (B6) Regulation of body fluid levels. (B7) Regulation of multicellular organismal process. (C1) Tumor necrosis factor production. (C2) System development. (C3) Negative regulation of tissue remodeling. (C4) Regulation of tissue remodeling. (C5) Regulation of angiogenesis. (D) Regulation of tumor necrosis factor production. (E) Regulation of tumor necrosis factor biosynthetic process. Similarly as Figure S1, the top level category, multicellular organismal process, labeled (A), was further mining down levels by levels into its subcategories labeled alphabetically with each letter for each down level and for each level, representative categories were further broken down into all its subcategories shown here. (PPT) [file pone.0043551.s004.ppt]

## Slide 1
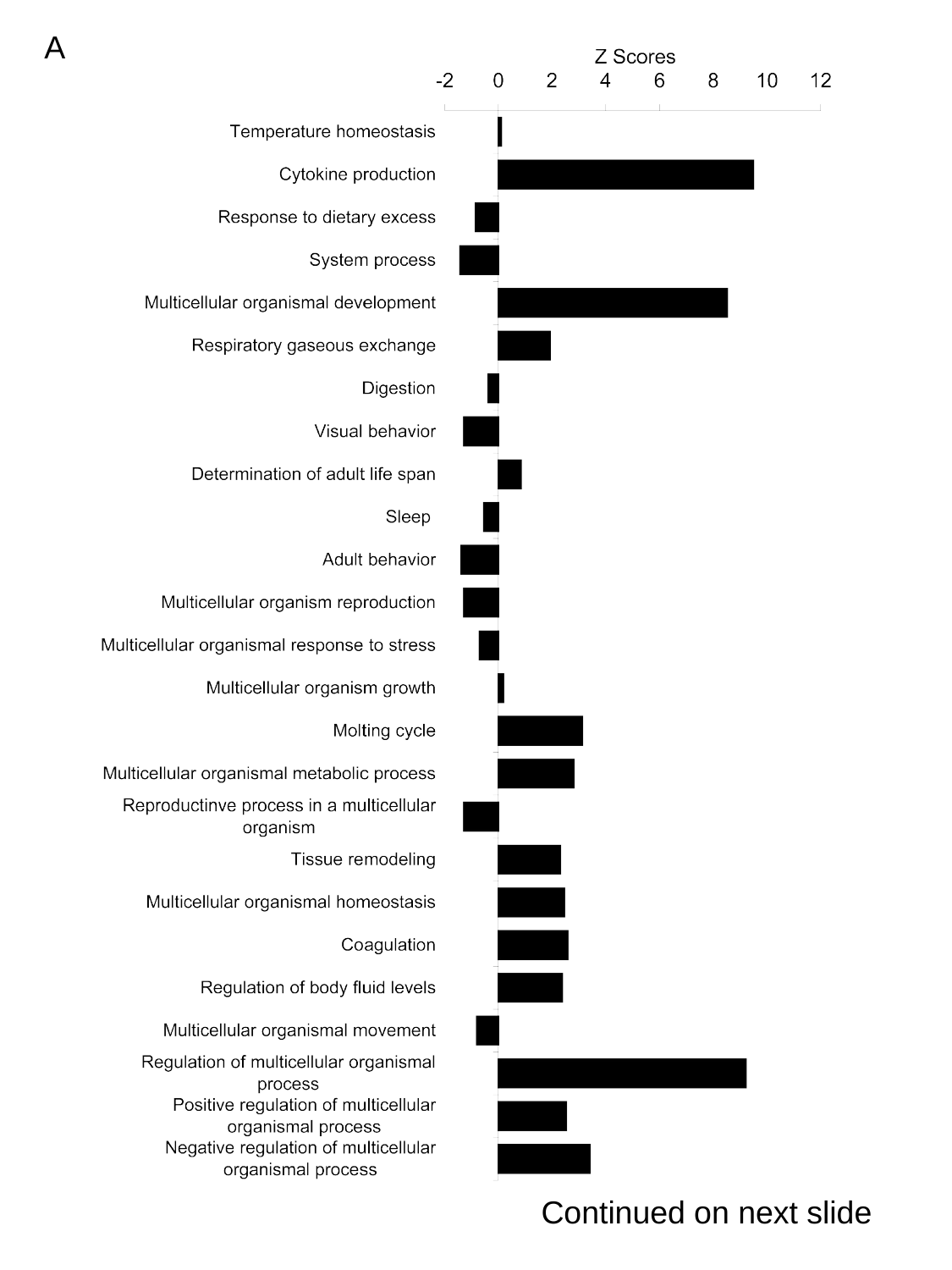

A
Continued on next slide

## Slide 2
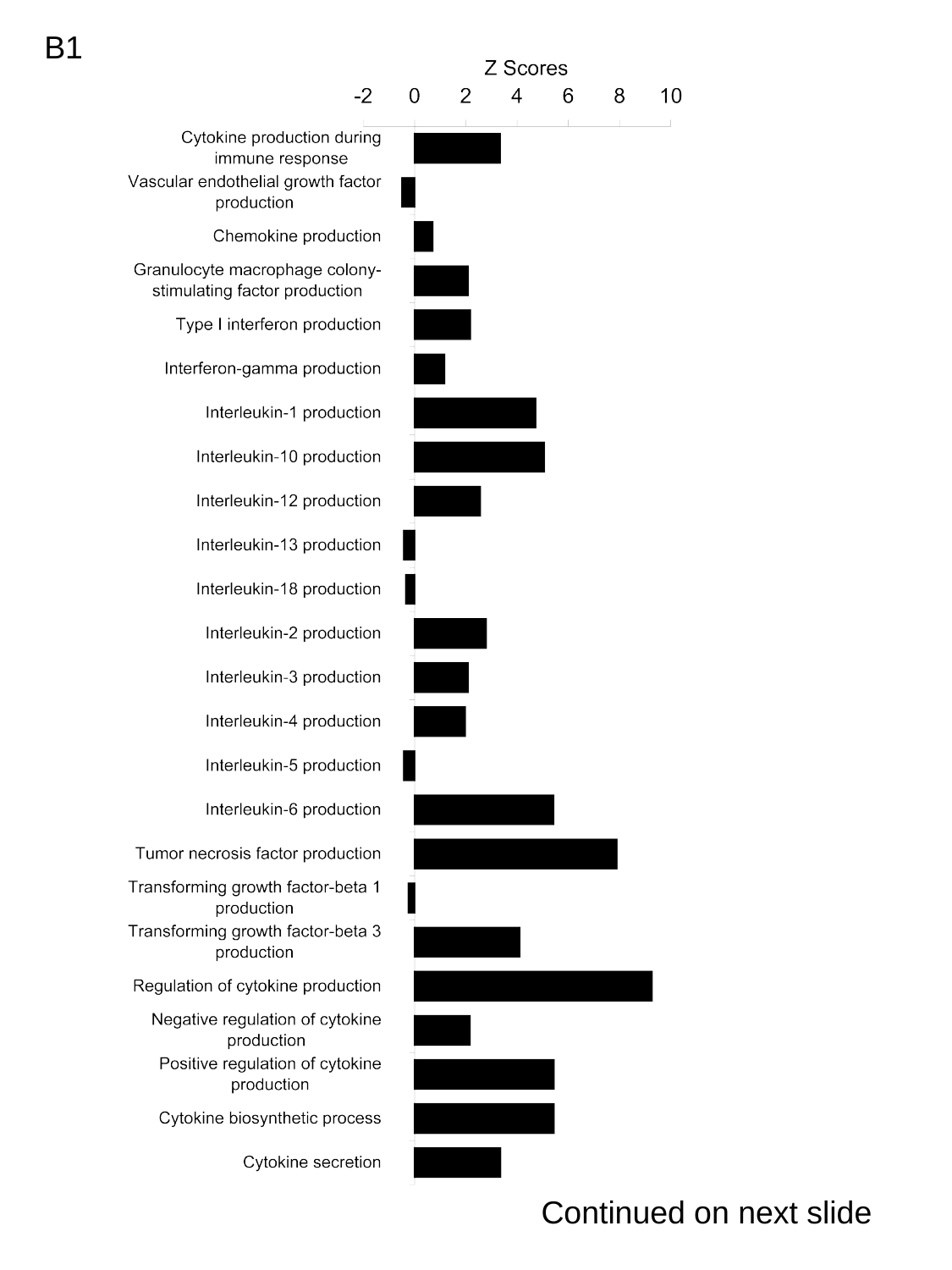

B1
Continued on next slide

## Slide 3
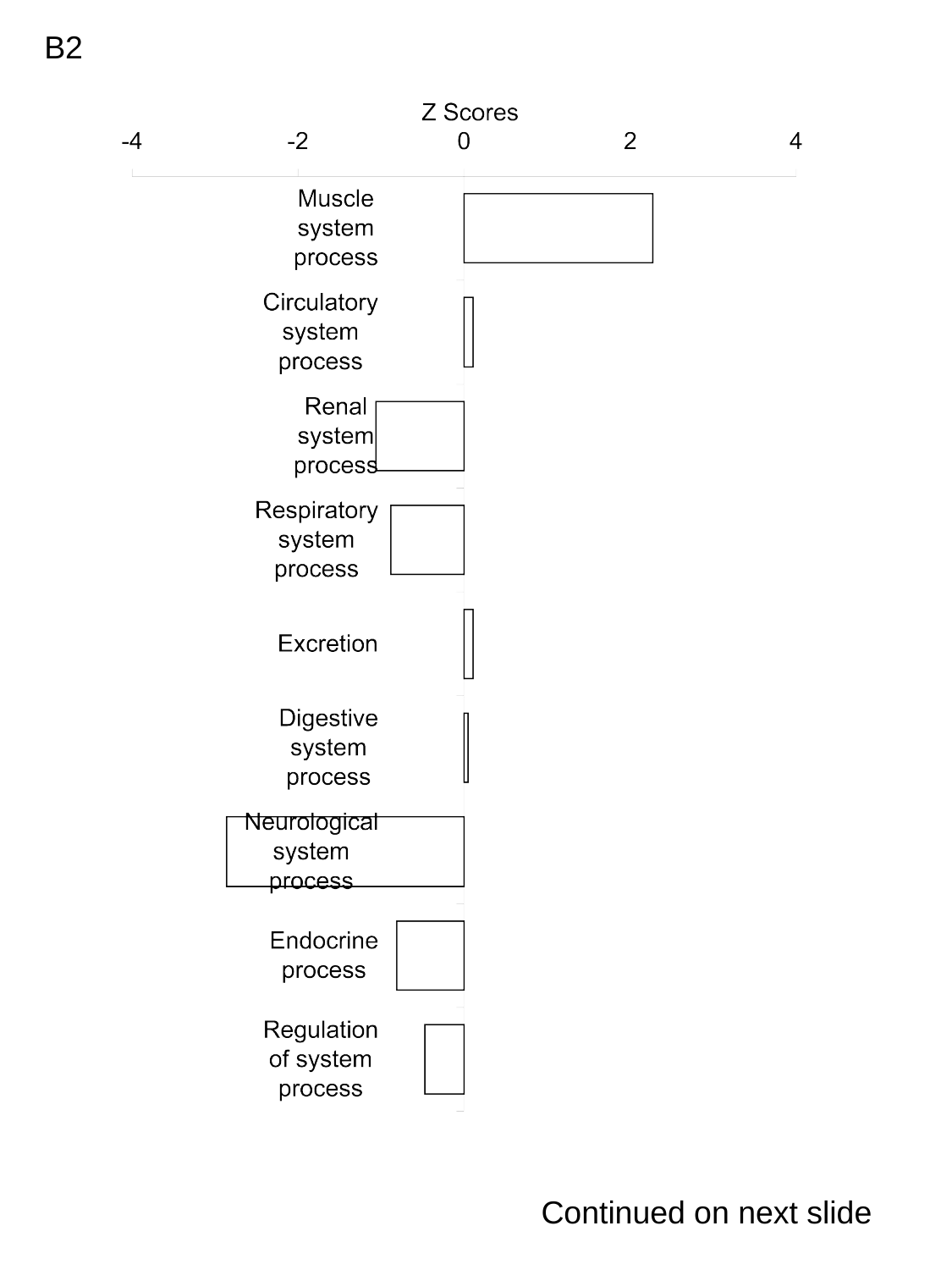

B2
Continued on next slide

## Slide 4
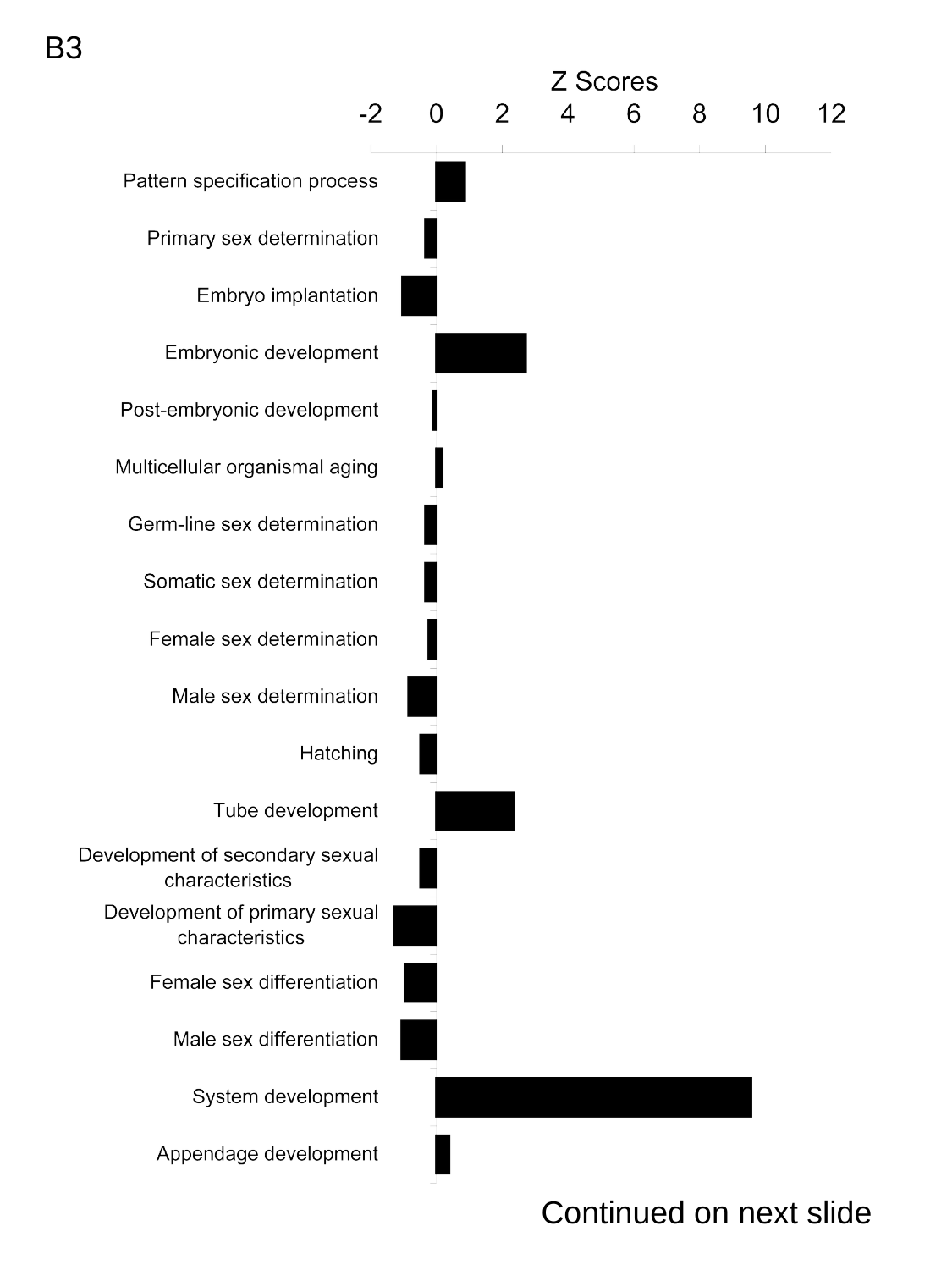

B3
Continued on next slide

## Slide 5
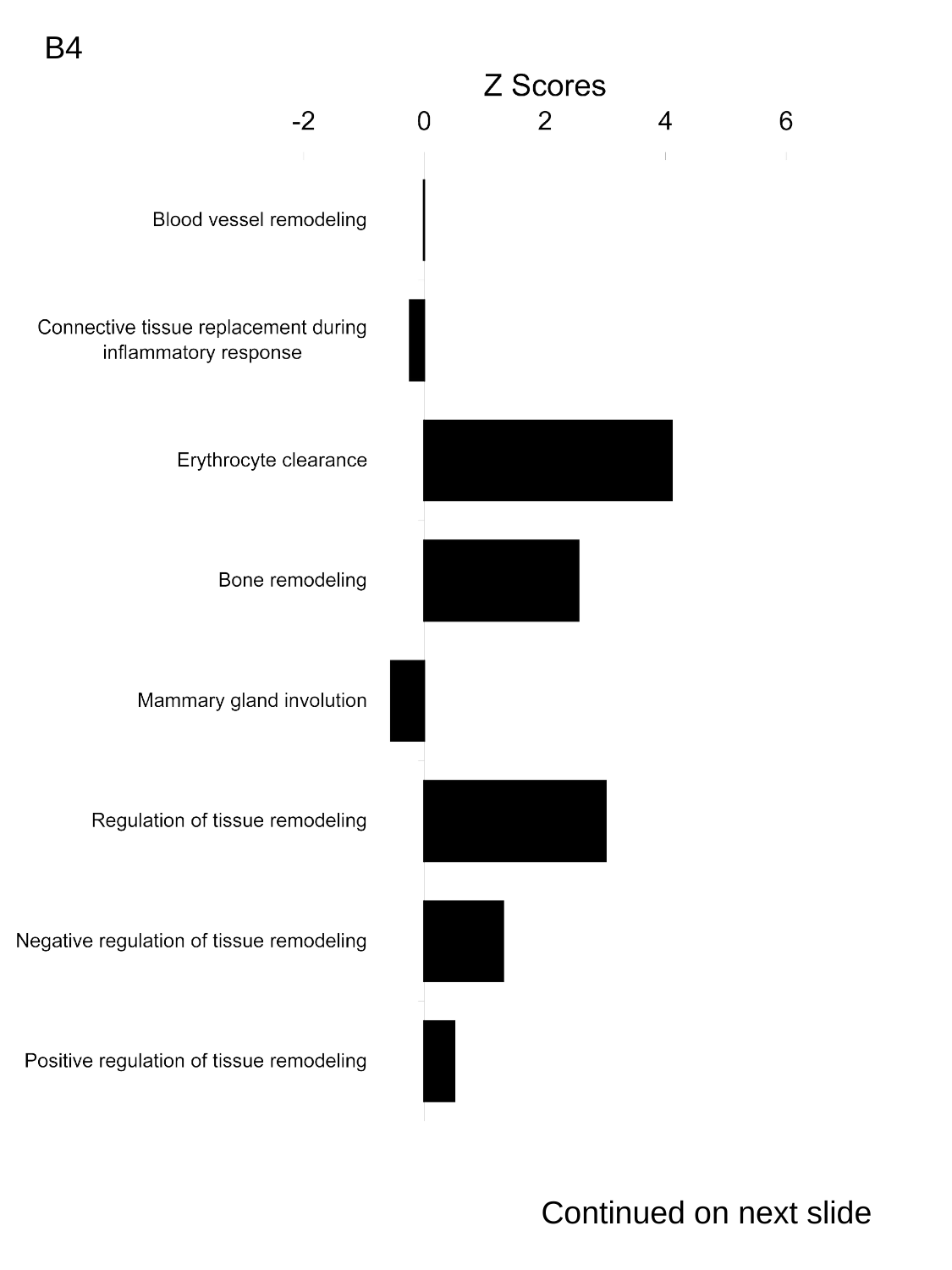

B4
Continued on next slide

## Slide 6
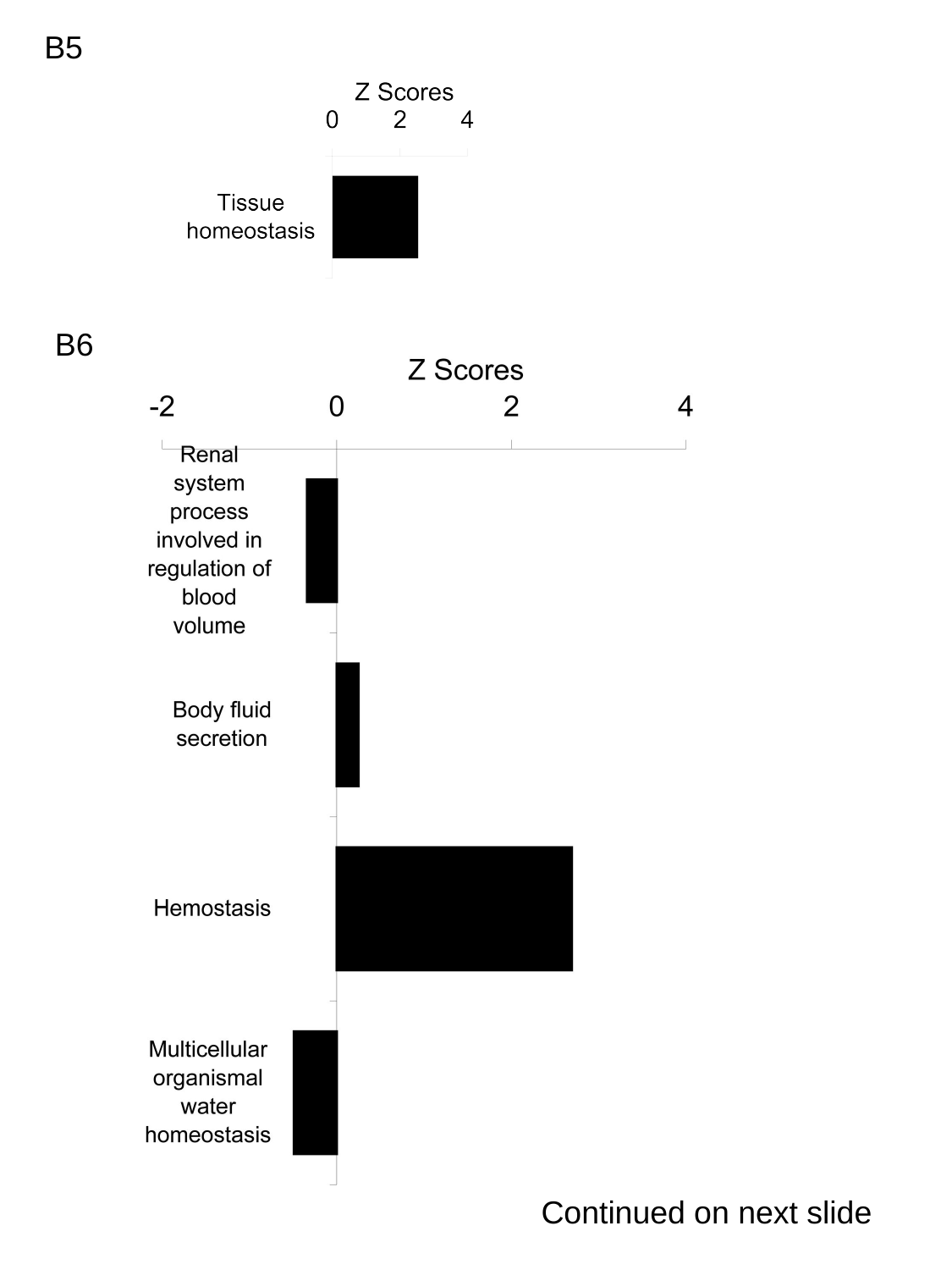

B5
B6
Continued on next slide

## Slide 7
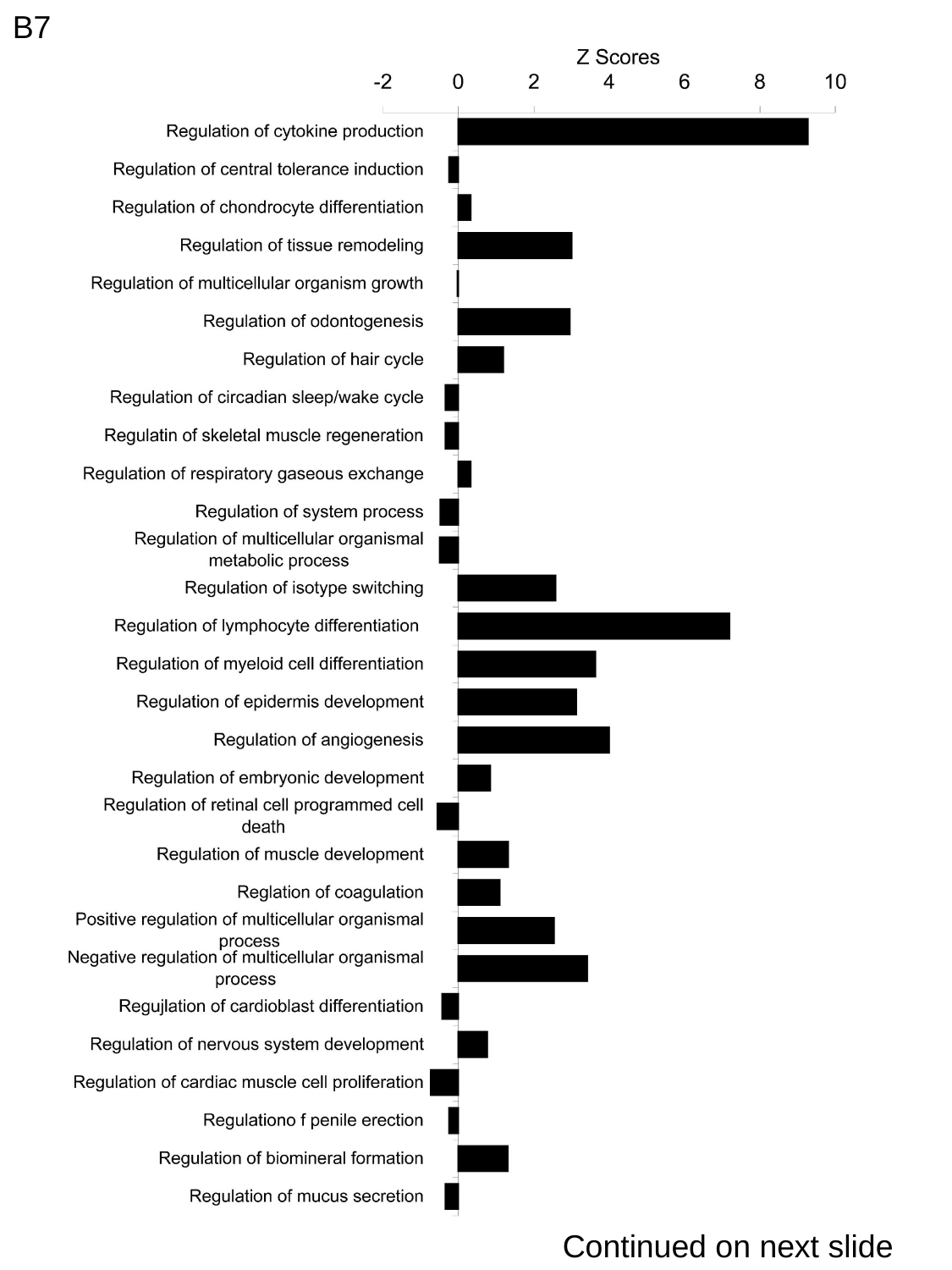

B7
Continued on next slide

## Slide 8
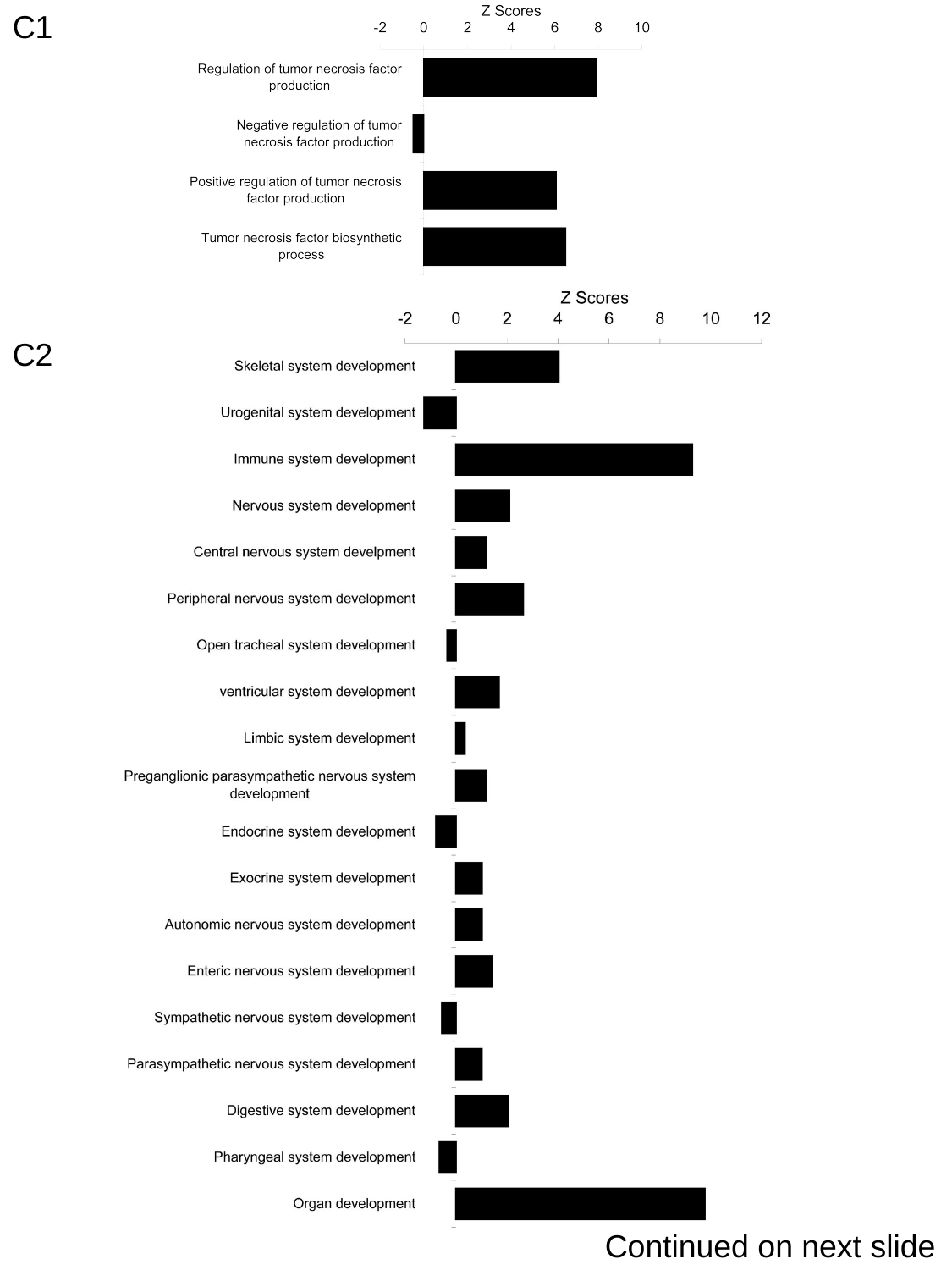

C1
C2
Continued on next slide

## Slide 9
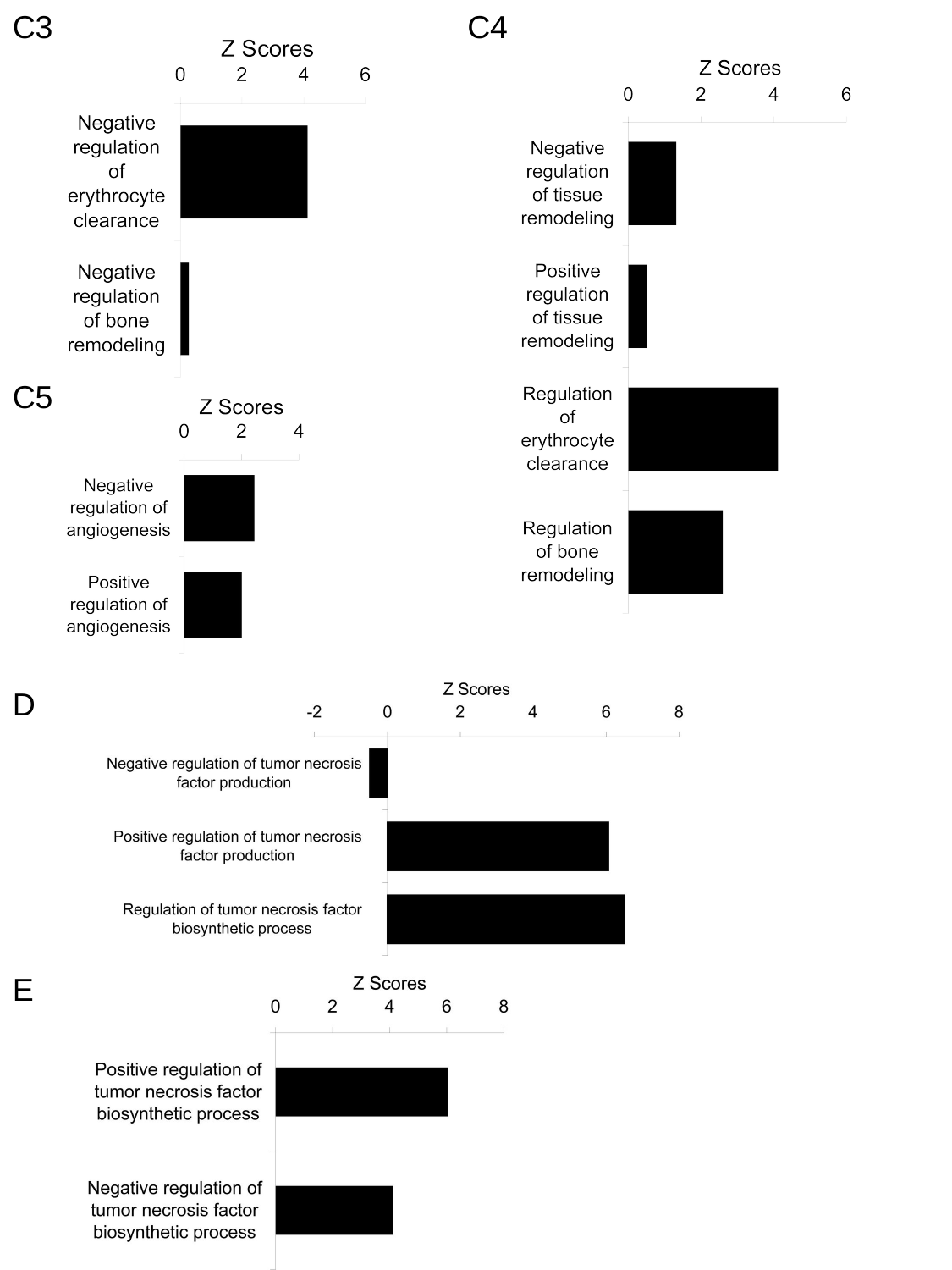

C3
C4
C5
D
E
