# Supplementary material for: Systems Analysis of a Mouse Xenograft Model Reveals Annexin A1 as a Regulator of Gene Expression in Tumor Stroma
Source: PLoS One. 2012 Oct 15;7(10):e43551. doi: 10.1371/journal.pone.0043551 (PMC3471933; doi:10.1371/journal.pone.0043551)
Supplement: Figure S5 — Breakdown of developmental process category into its subcategories. (A) Developmental process. (B1) Multicellular organismal development. (B2) Anatomical structure morphogenesis. (B3) Anatomical structure formation. (B4) Anatomical structure development. (B5) Cellular developmental process. (B6) Regulation of developmental process. (B7) Negative regulation of developmental process. (B8) Positive regulation of developmental process. (C) Positive regulation of programmed cell death. (D1) Induction of programmed cell death. (D2) Positive Regulation of apoptosis. (E1) Induction of apoptosis. (E2) Positive regulation of lymphocyte apoptosis. (F1) Induction of apoptosis by extracellular signals. (F2) Induction of apoptosis by intracellular signals. Similarly as Figure S1, the top level category, developmental process, labeled (A), was further mining down levels by levels into its subcategories labeled alphabetically with each letter for each down level and for each level, representative categories were further broken down into all its subcategories shown here. (PPT) [file pone.0043551.s005.ppt]

## Slide 1
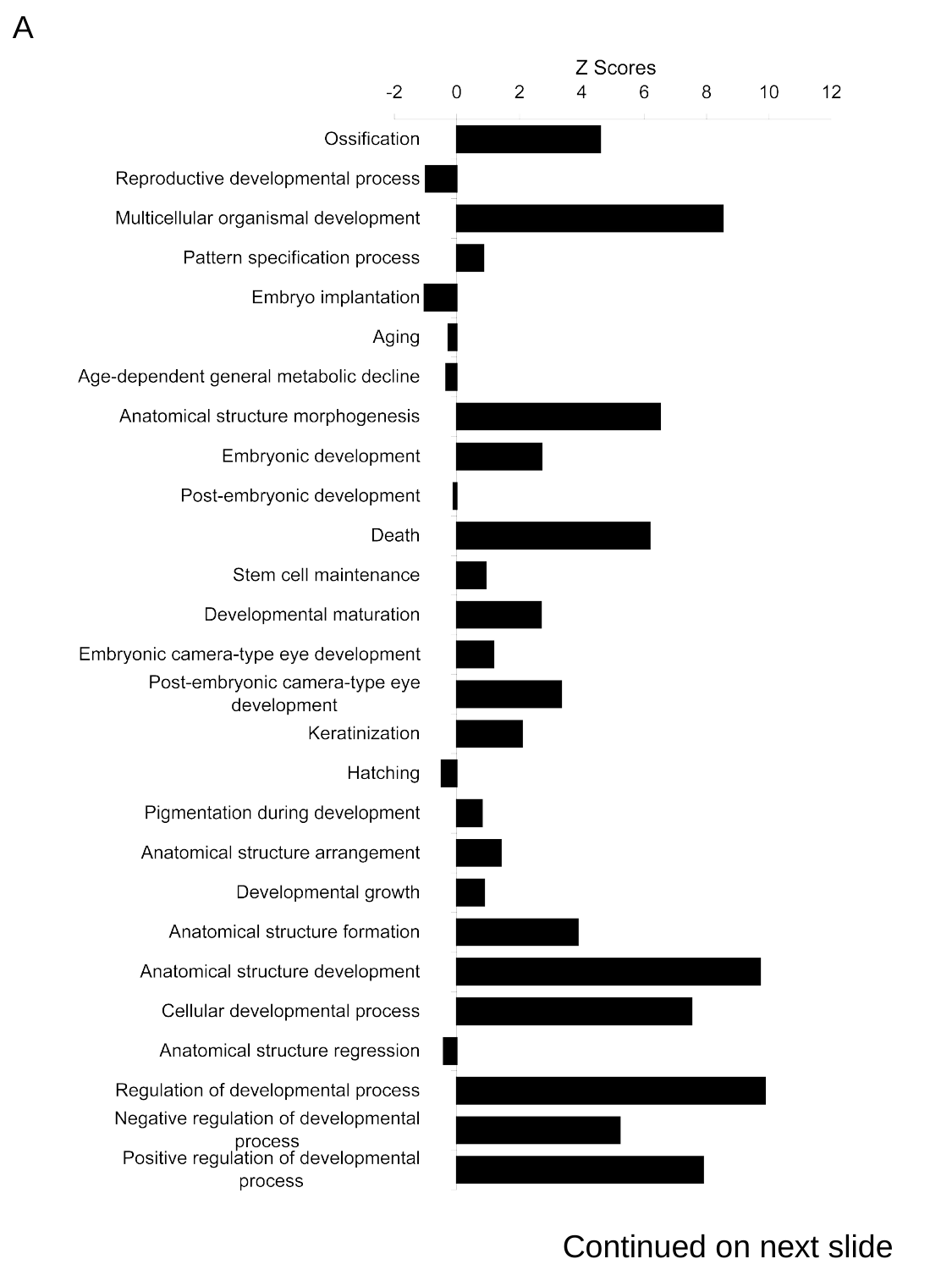

A
Continued on next slide

## Slide 2
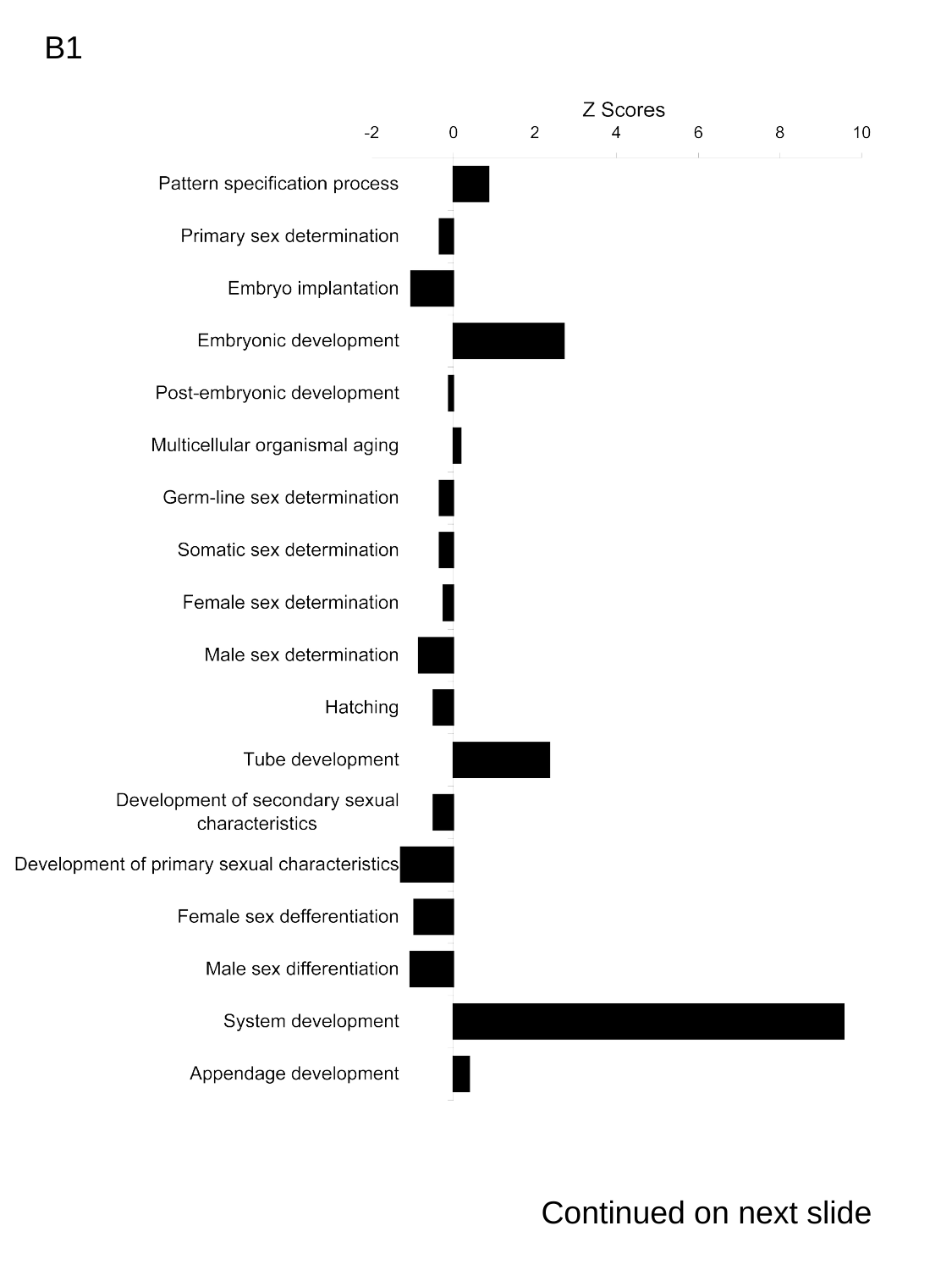

B1
Continued on next slide

## Slide 3
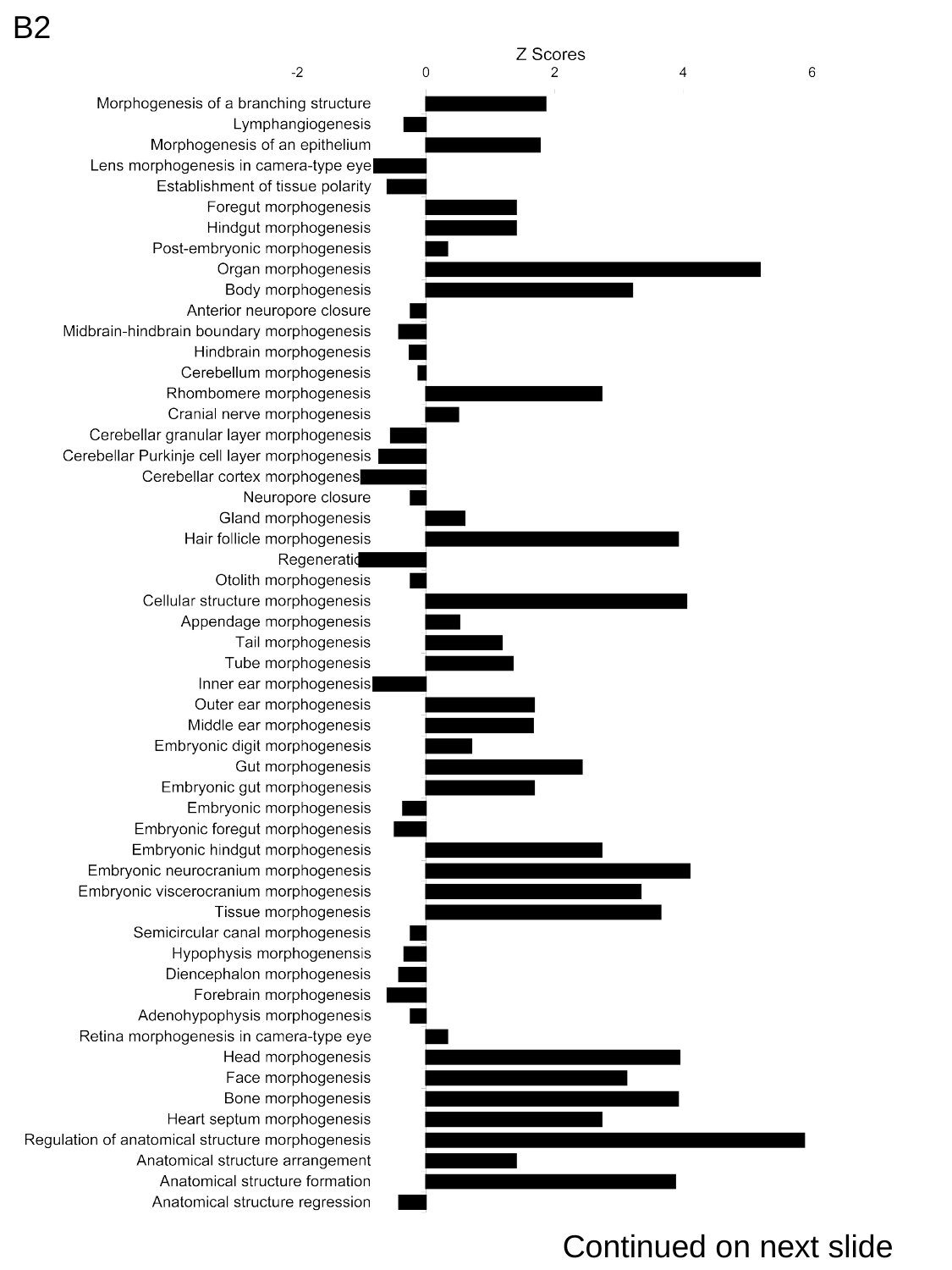

B2
Continued on next slide

## Slide 4
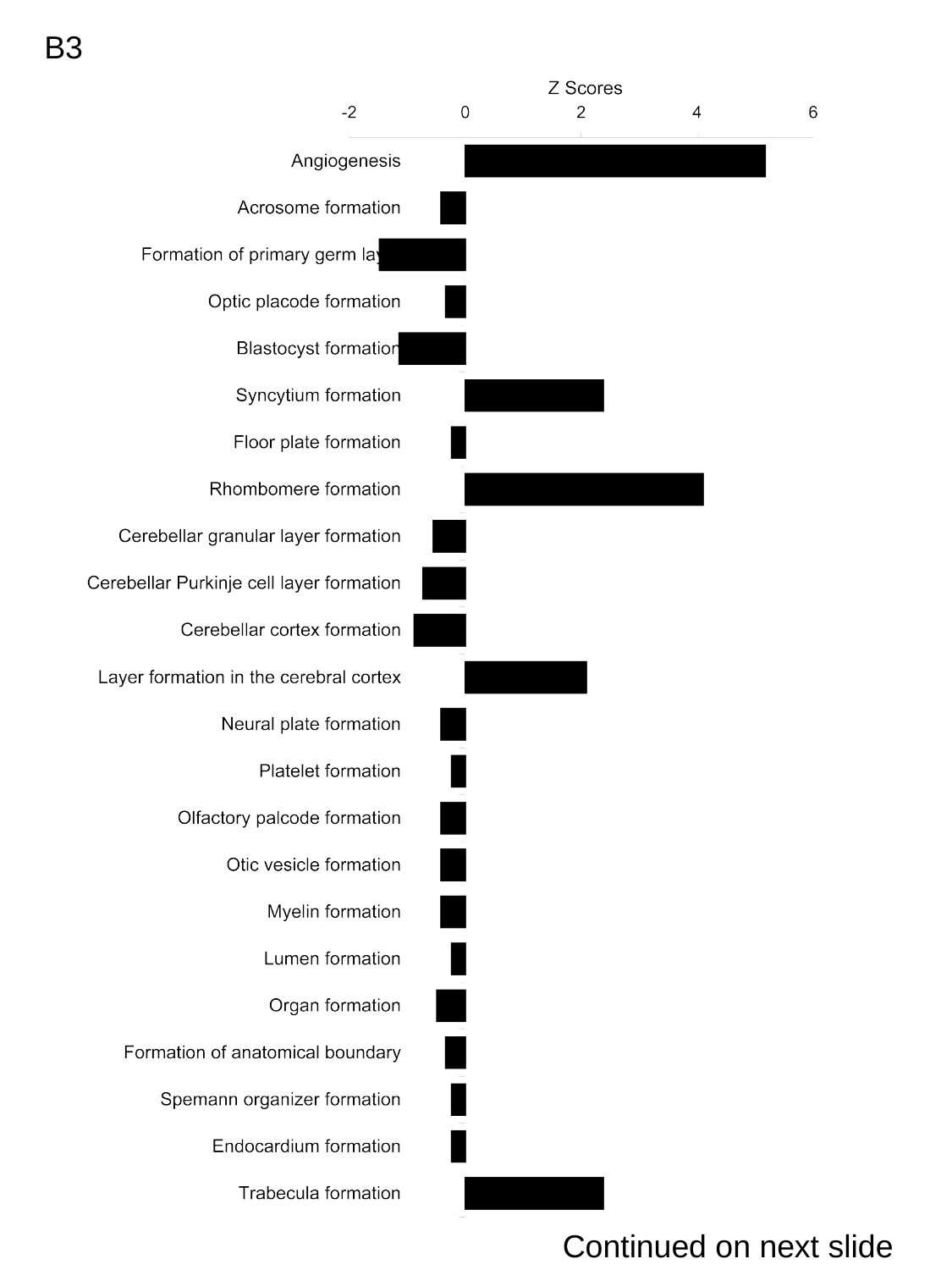

B3
Continued on next slide

## Slide 5
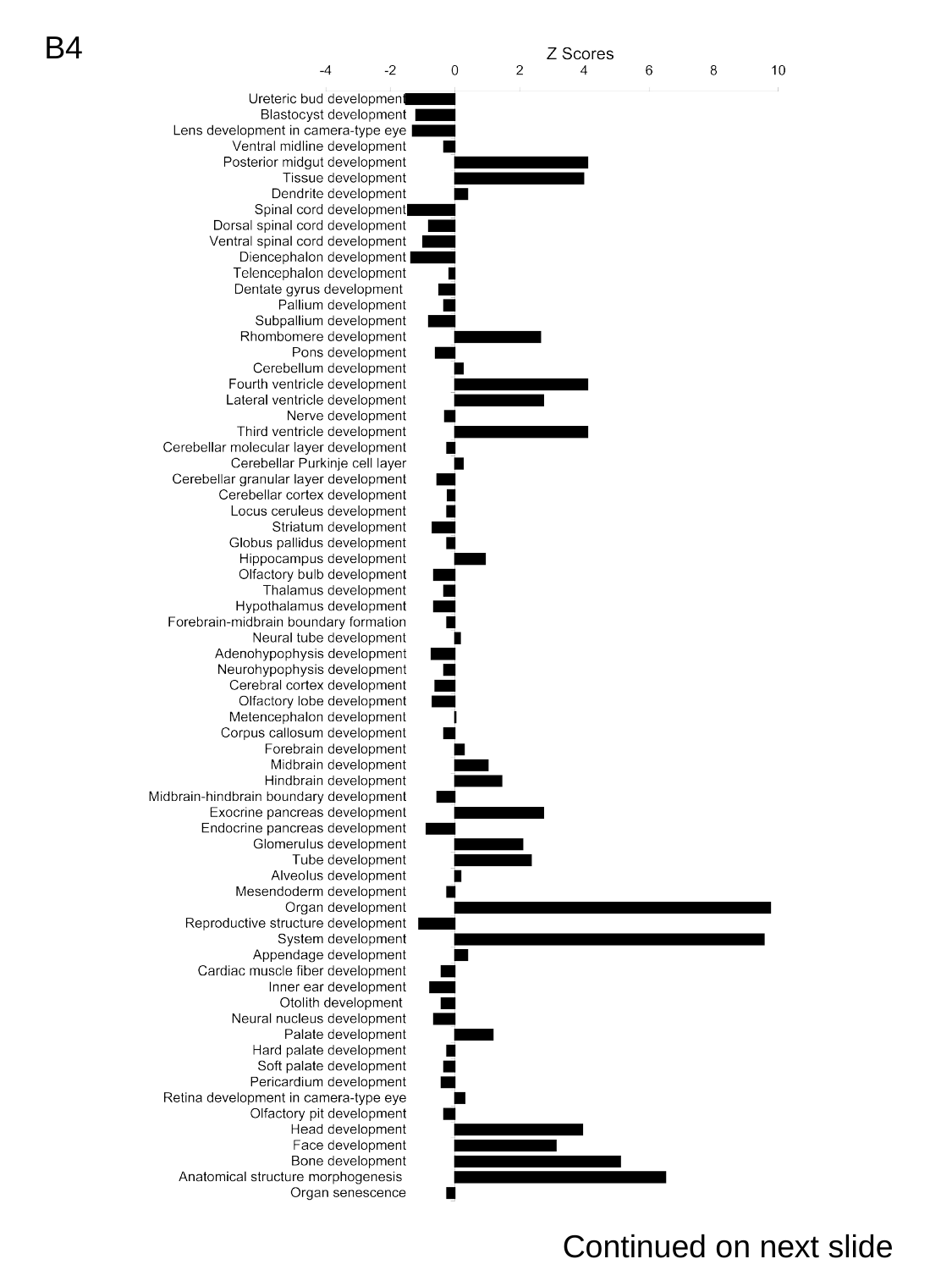

B4
Continued on next slide

## Slide 6
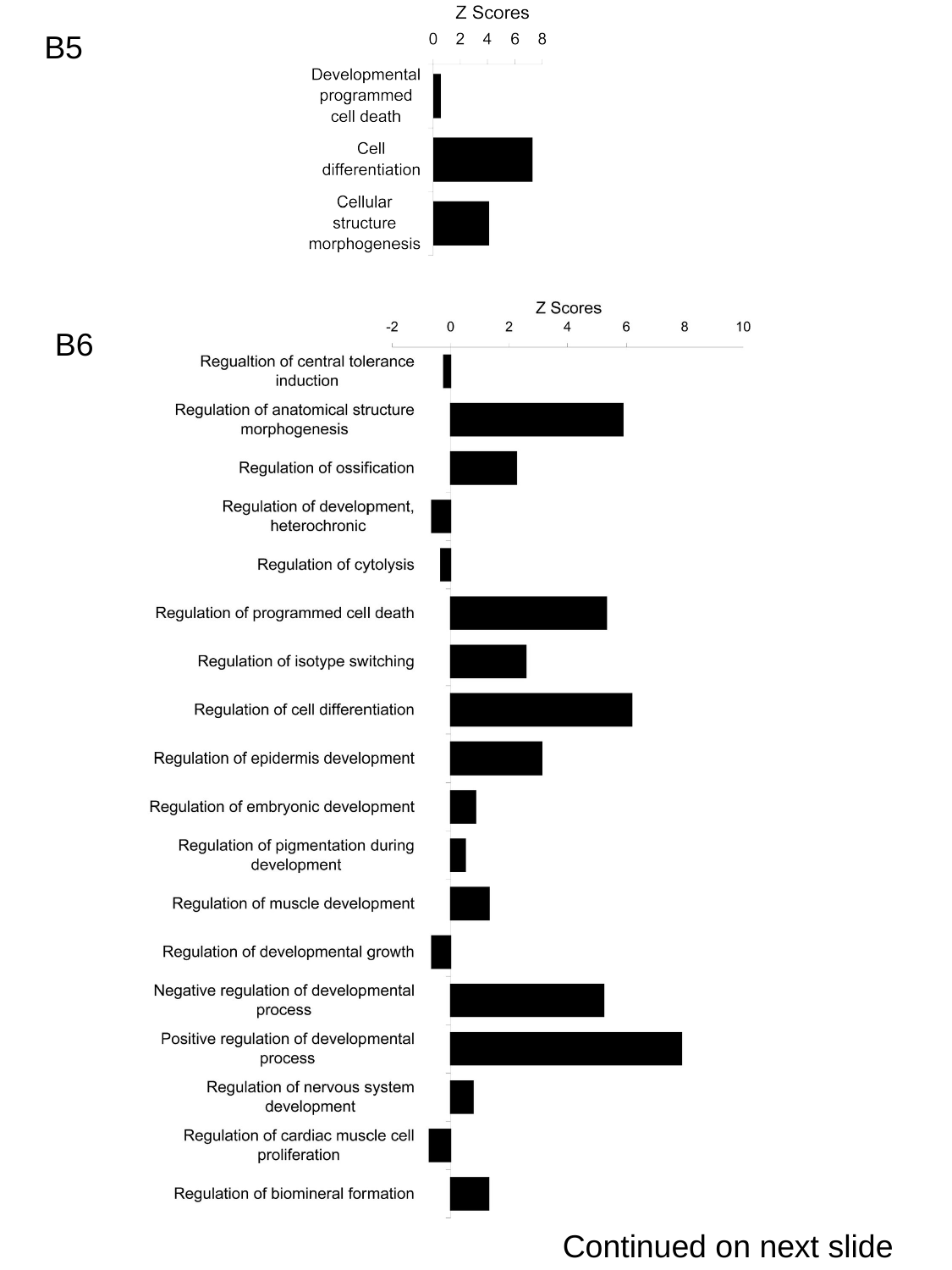

B5
B6
Continued on next slide

## Slide 7
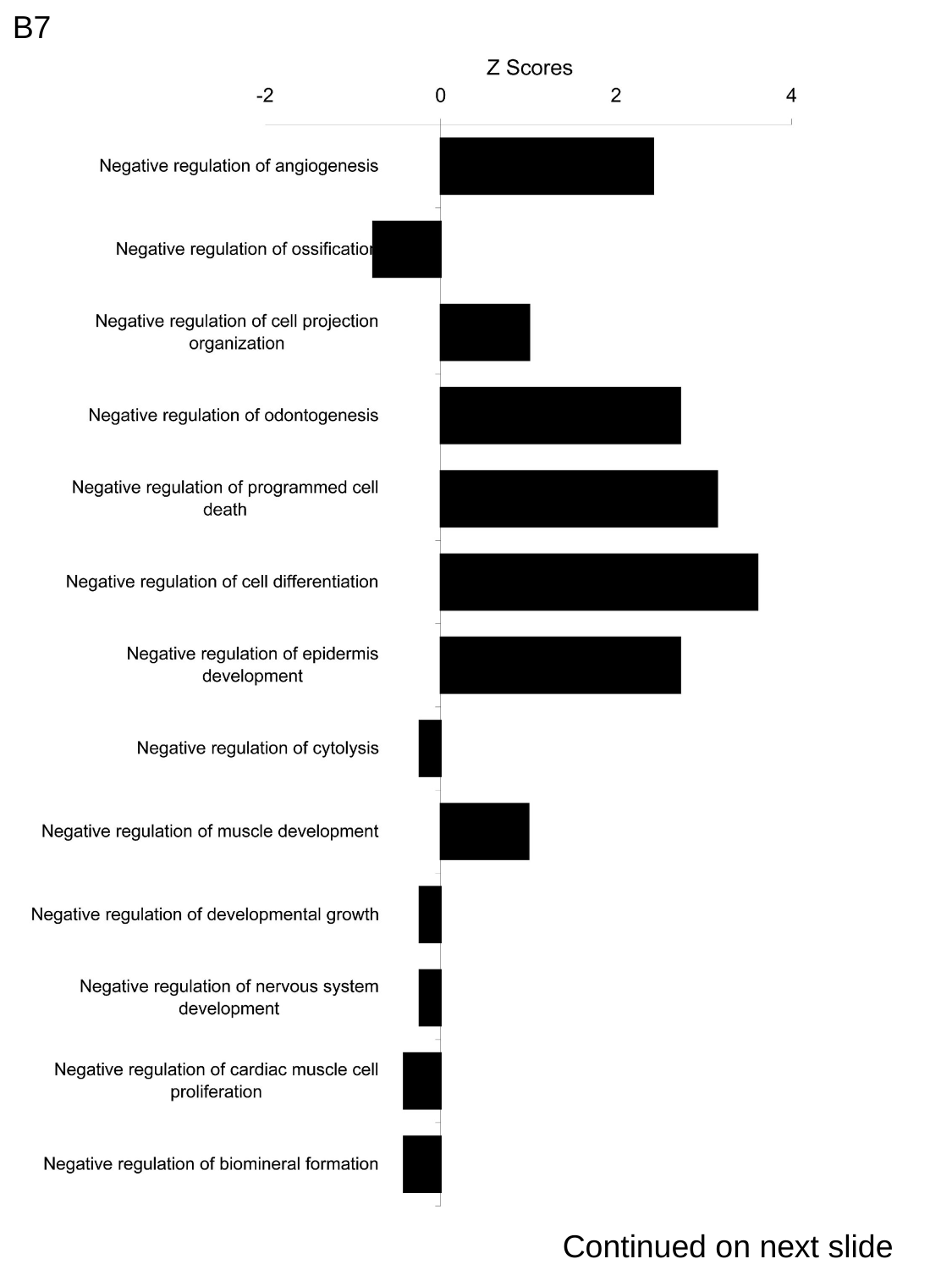

B7
Continued on next slide

## Slide 8
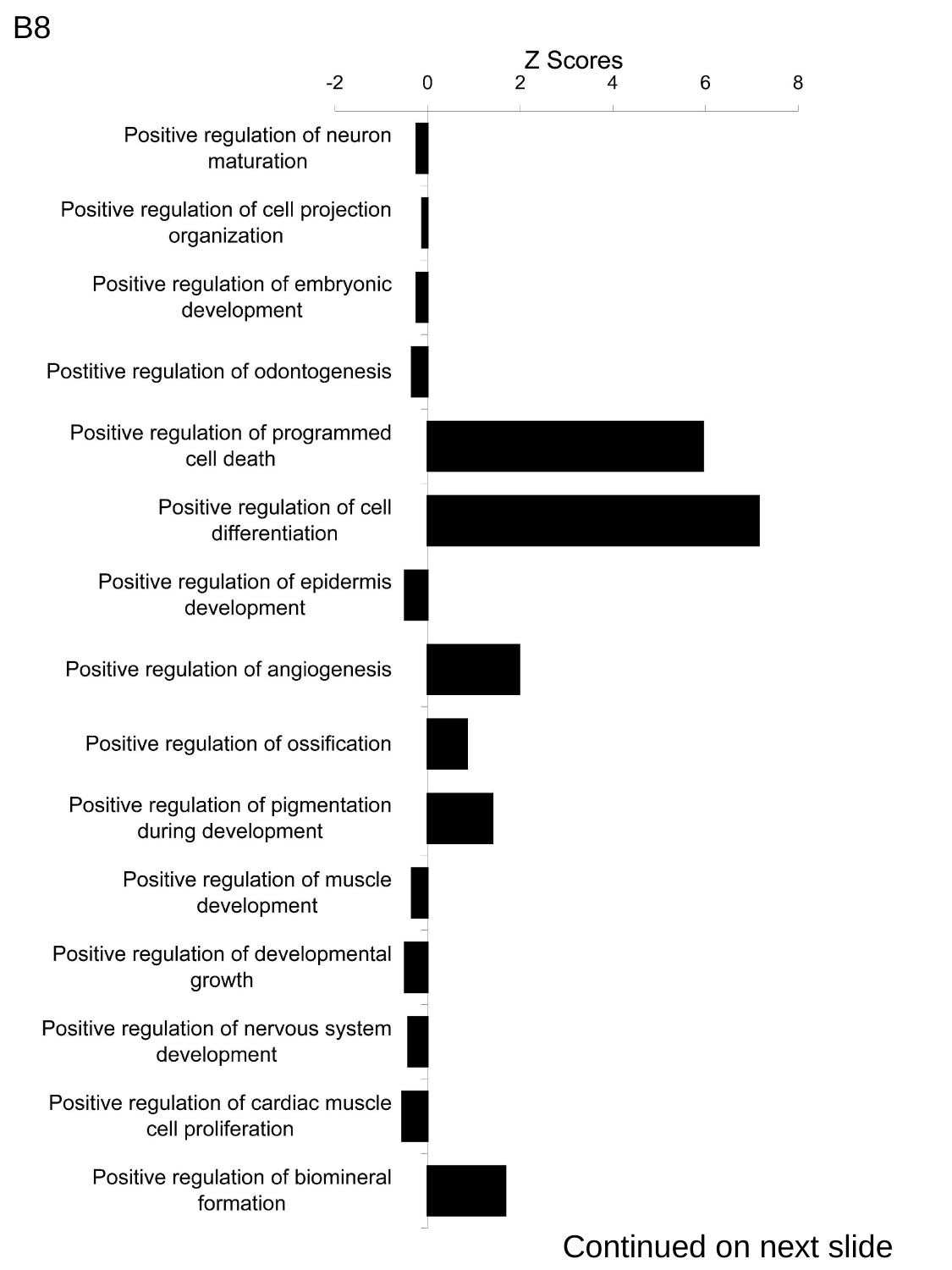

B8
Continued on next slide

## Slide 9
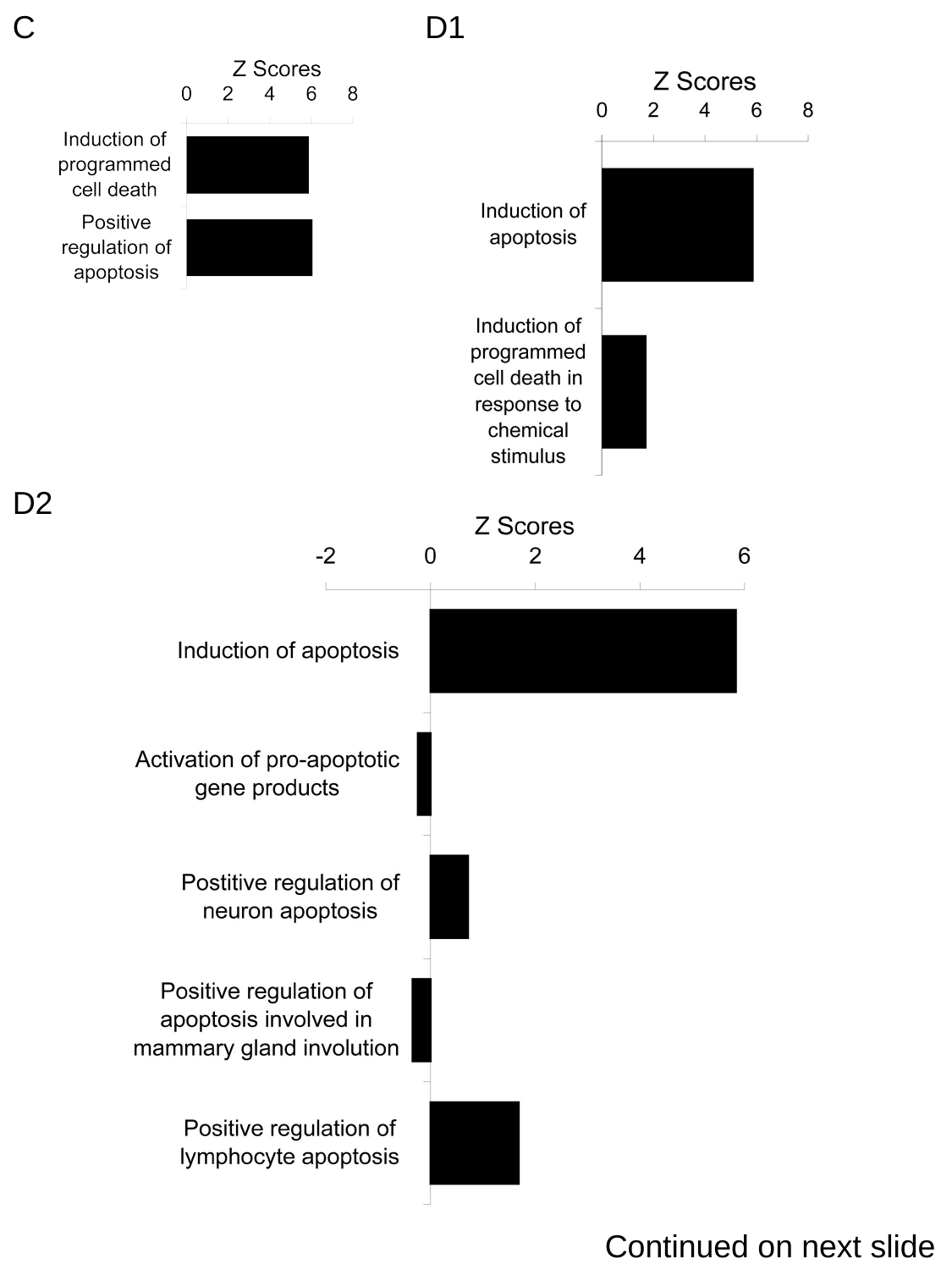

C
D1
D2
Continued on next slide

## Slide 10
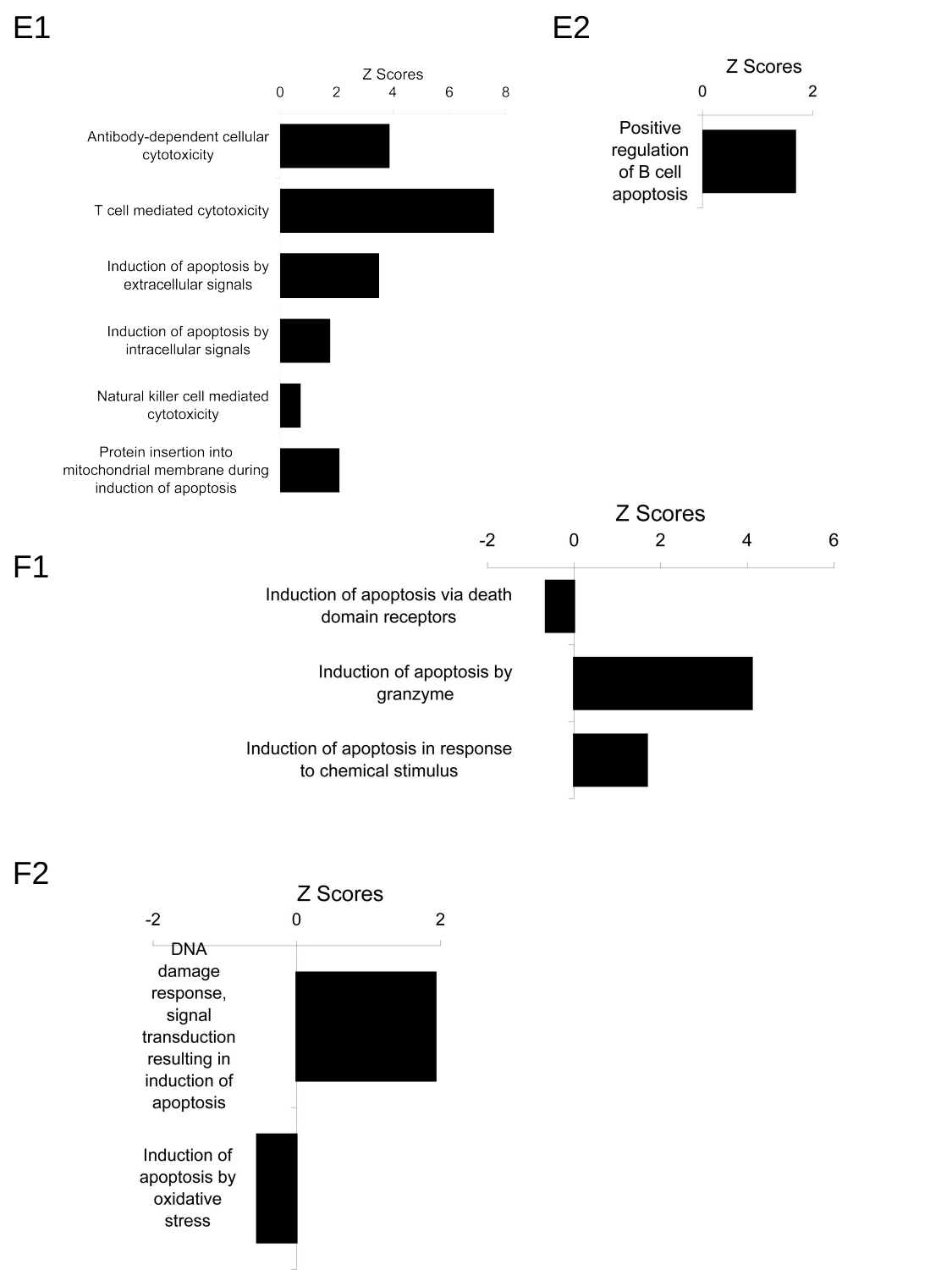

E1
E2
F1
F2
