# Supplementary material for: Systems Analysis of a Mouse Xenograft Model Reveals Annexin A1 as a Regulator of Gene Expression in Tumor Stroma
Source: PLoS One. 2012 Oct 15;7(10):e43551. doi: 10.1371/journal.pone.0043551 (PMC3471933; doi:10.1371/journal.pone.0043551)
Supplement: Figure S7 — Breakdown of cellular process category into its subcategories. (A) Cellular process. (B1) Cell activation. (B2) Cell motion. (B3) Cell communication. (B4) Cell adhesion. (B5) Cell proliferation. (B6) Cellular component organization. (B7) Cellular developmental process. (C1) Mononuclear cell proliferation. (C2) Cell differentiation. (D1) Lymphocyte proliferation. (D2) Leukocyte differentiation. (E) Myeloid leukocyte differentiation. Similarly as Figure S1, the top level category, cellular process, labeled (A), was further mining down levels by levels into its subcategories labeled alphabetically with each letter for each down level and for each level, representative categories were further broken down into all its subcategories shown here. (PPT) [file pone.0043551.s007.ppt]

## Slide 1
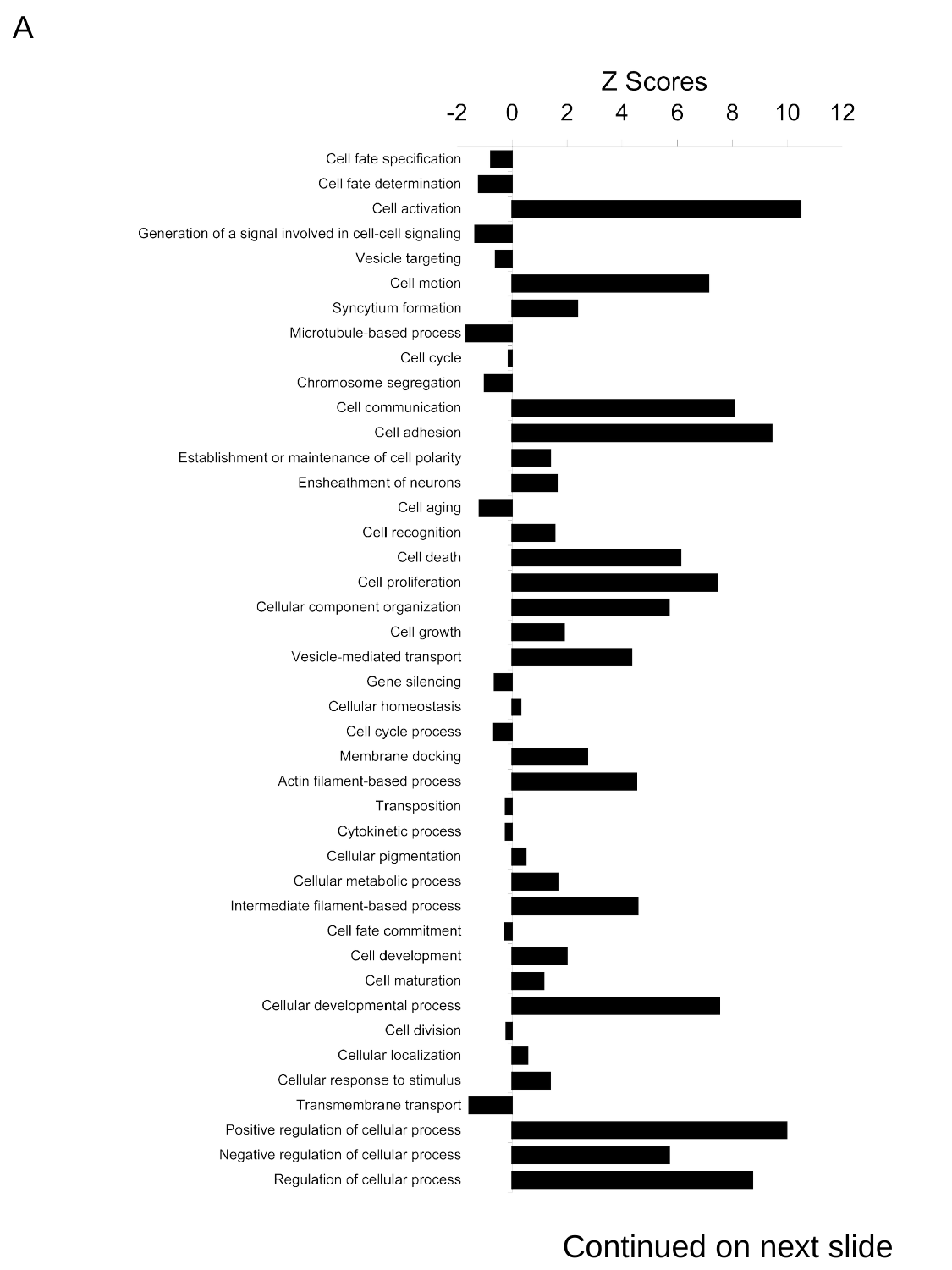

A
Continued on next slide

## Slide 2
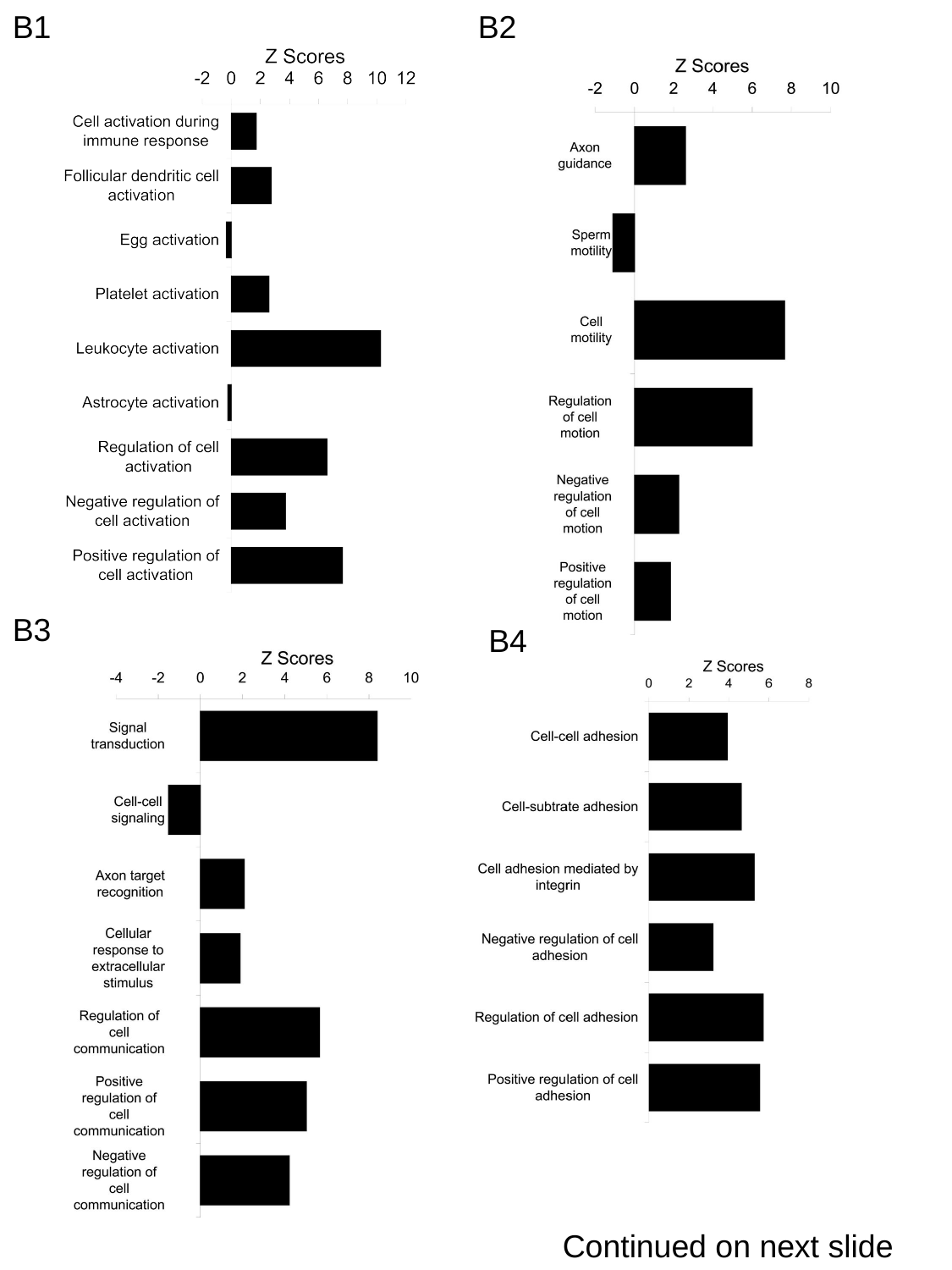

B1
B2
B3
B4
Continued on next slide

## Slide 3
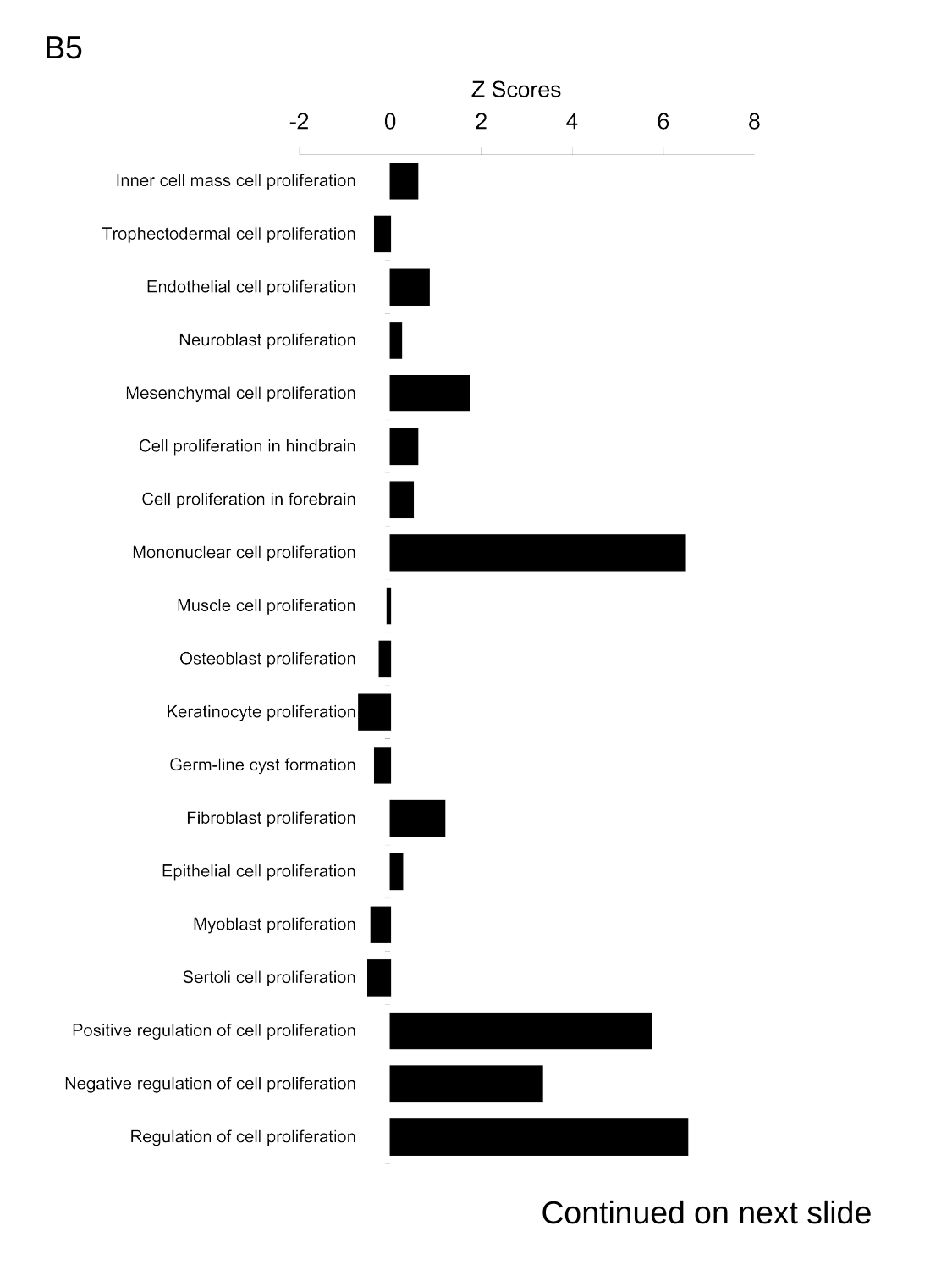

B5
Continued on next slide

## Slide 4
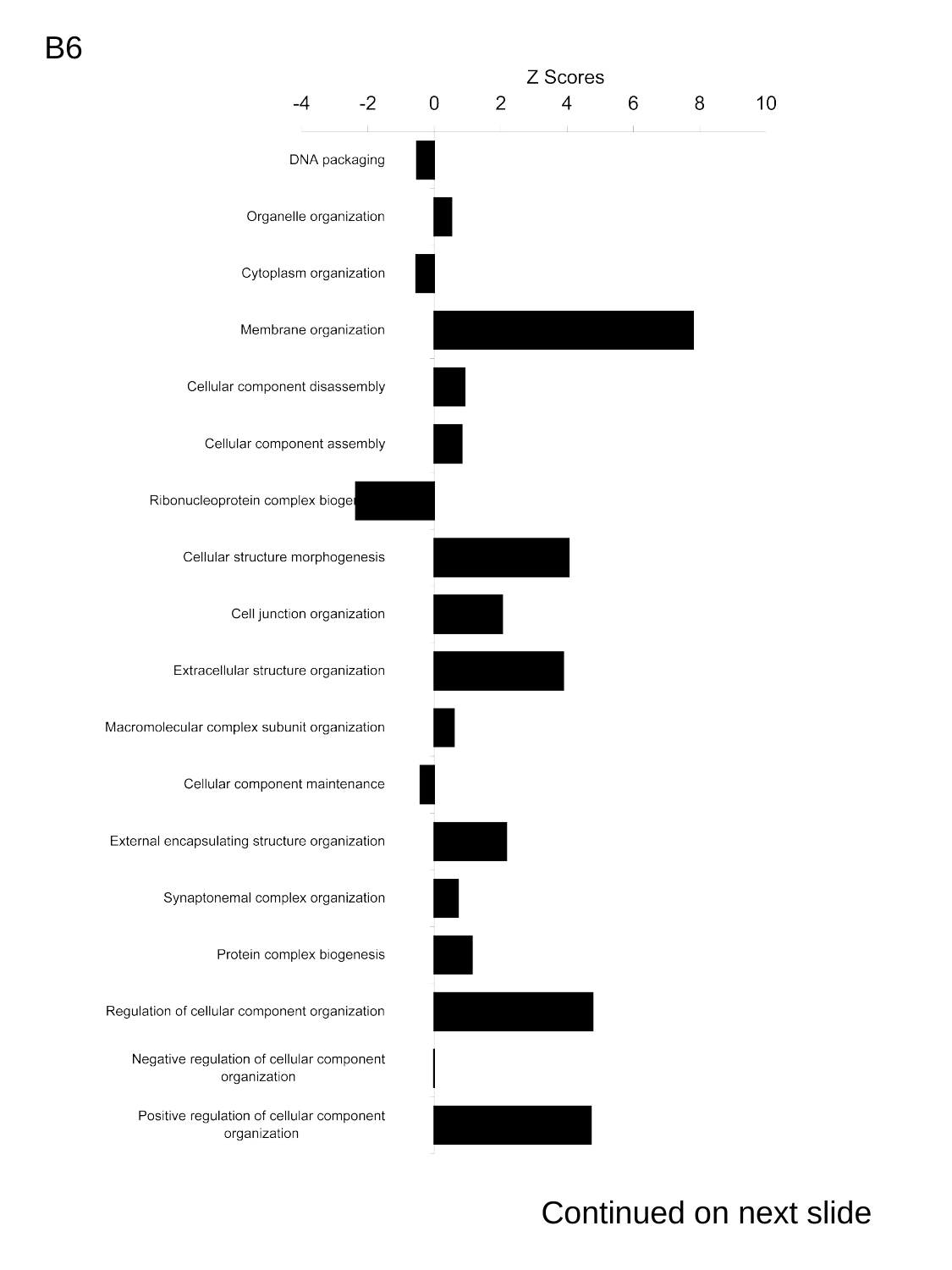

B6
Continued on next slide

## Slide 5
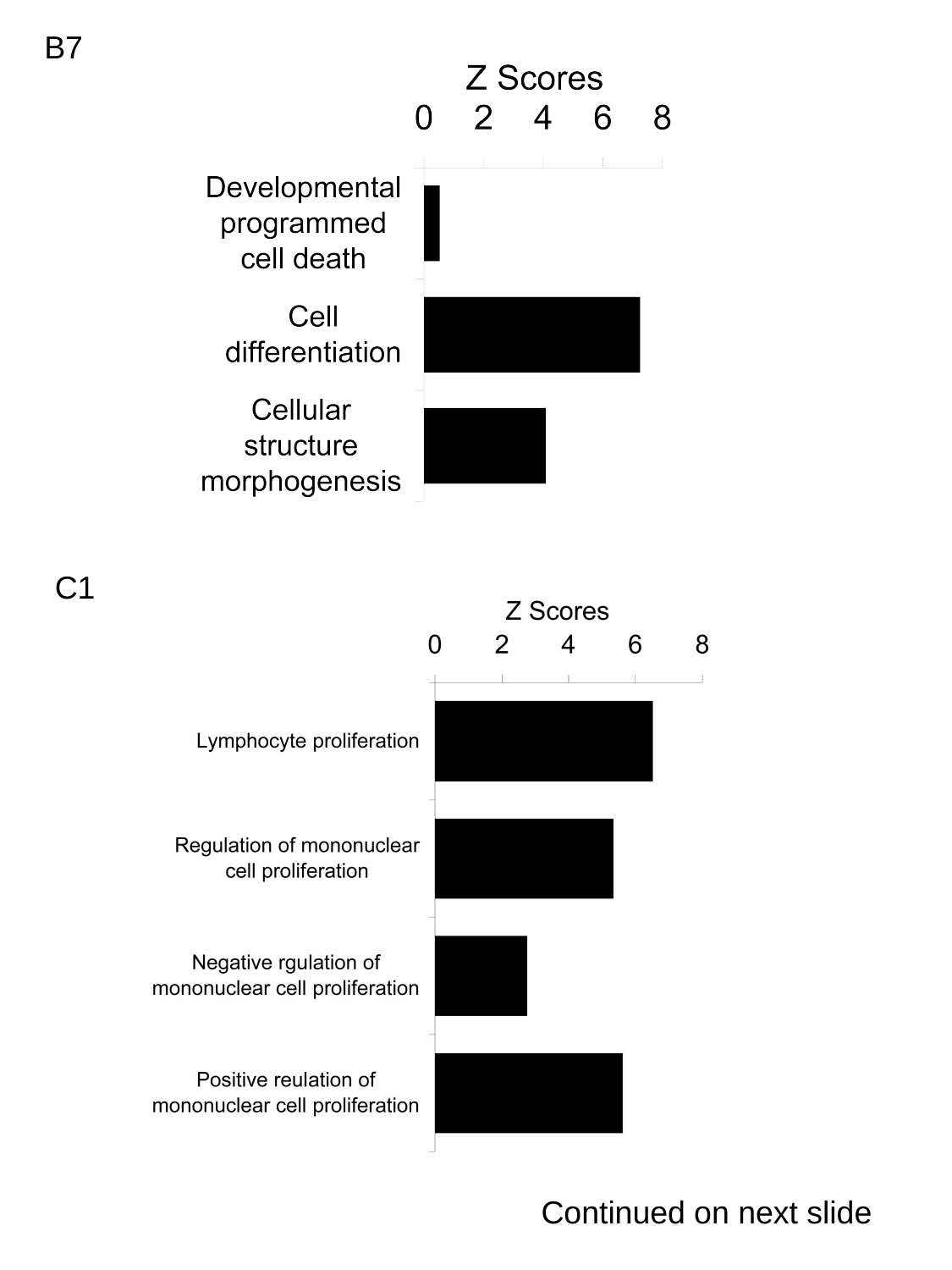

B7
C1
Continued on next slide

## Slide 6
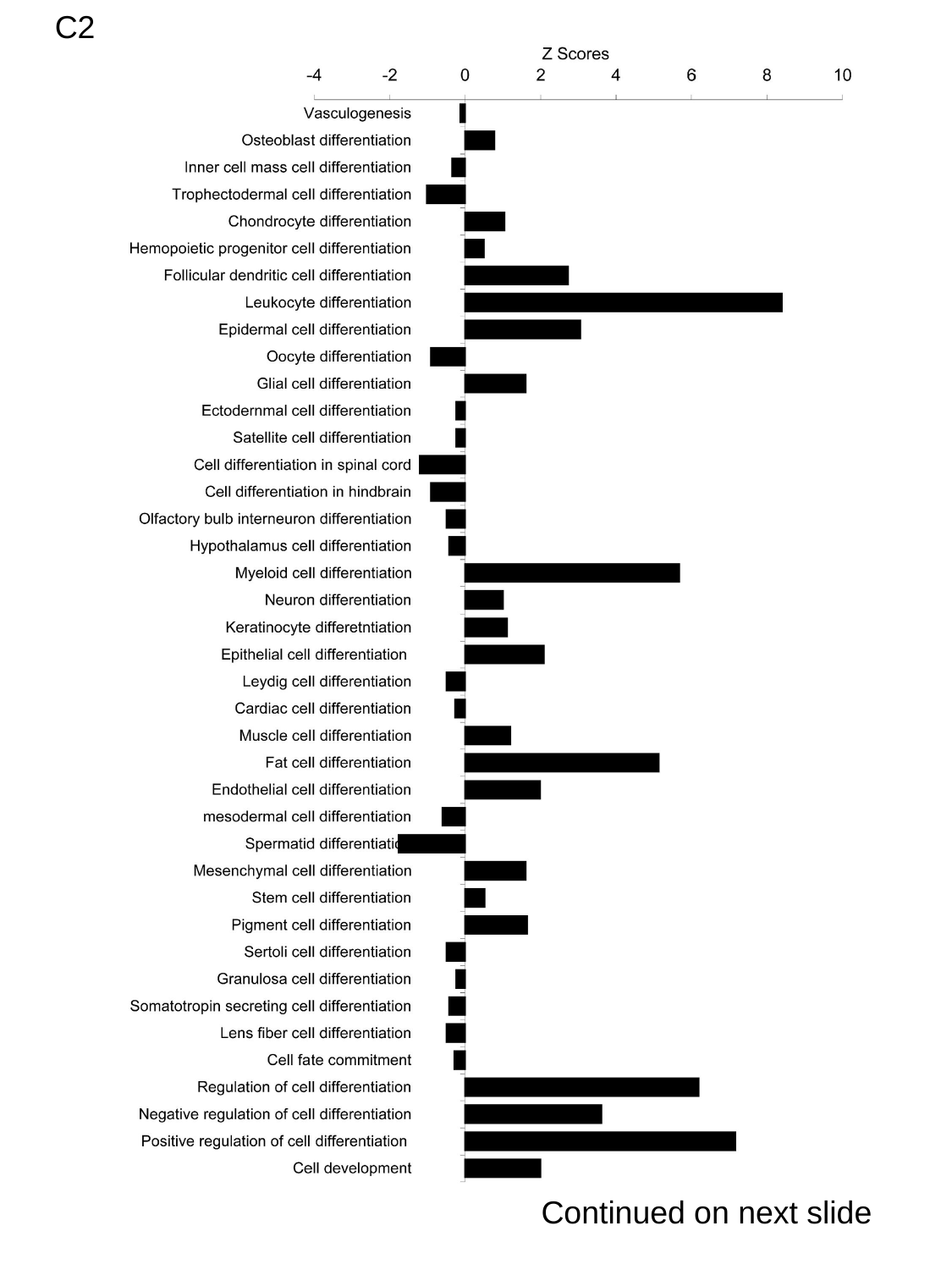

C2
Continued on next slide

## Slide 7
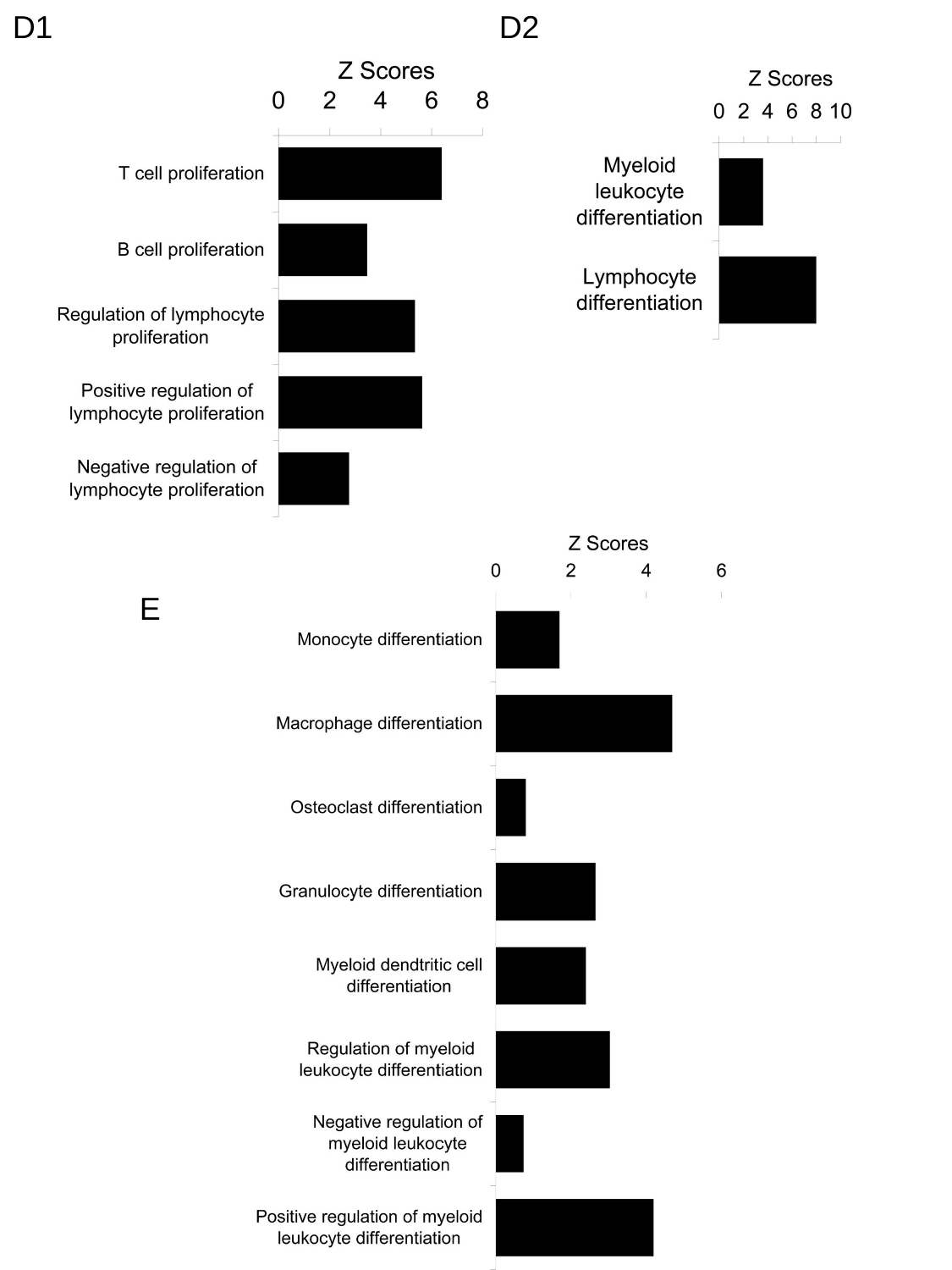

D1
D2
E
