# Supplementary material for: Systems Analysis of a Mouse Xenograft Model Reveals Annexin A1 as a Regulator of Gene Expression in Tumor Stroma
Source: PLoS One. 2012 Oct 15;7(10):e43551. doi: 10.1371/journal.pone.0043551 (PMC3471933; doi:10.1371/journal.pone.0043551)
Supplement: Figure S8 — Breakdown of response to stimulus category into its subcategories. (A) Response to stimulus. (B1) Response to external stimulus. (B2) Response to stress. (C1) Defense response. (C2) Response to wounding. (C3) Taxis. (D1) Inflammatory response. (D2) Chemotaxis. (E) Acute inflammatory response. (F) Activation of plasma proteins during acute inflammatory response. (G) Complement activation. Similarly as Figure S1, the top level category, response to stimulus, labeled (A), was further mining down levels by levels into its subcategories labeled alphabetically with each letter for each down level and for each level, representative categories were further broken down into all its subcategories shown here. (PPT) [file pone.0043551.s008.ppt]

## Slide 1
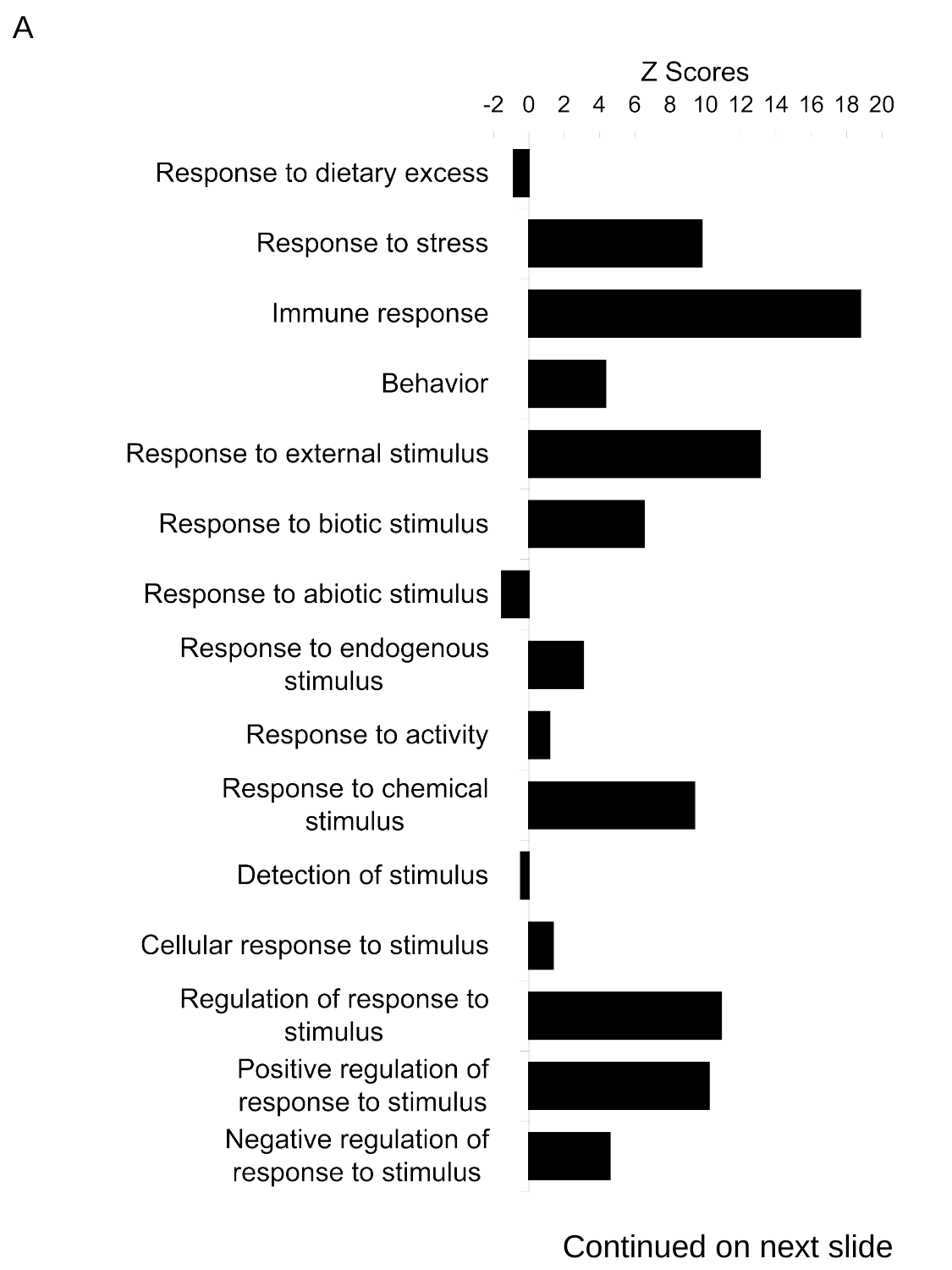

A
Continued on next slide

## Slide 2
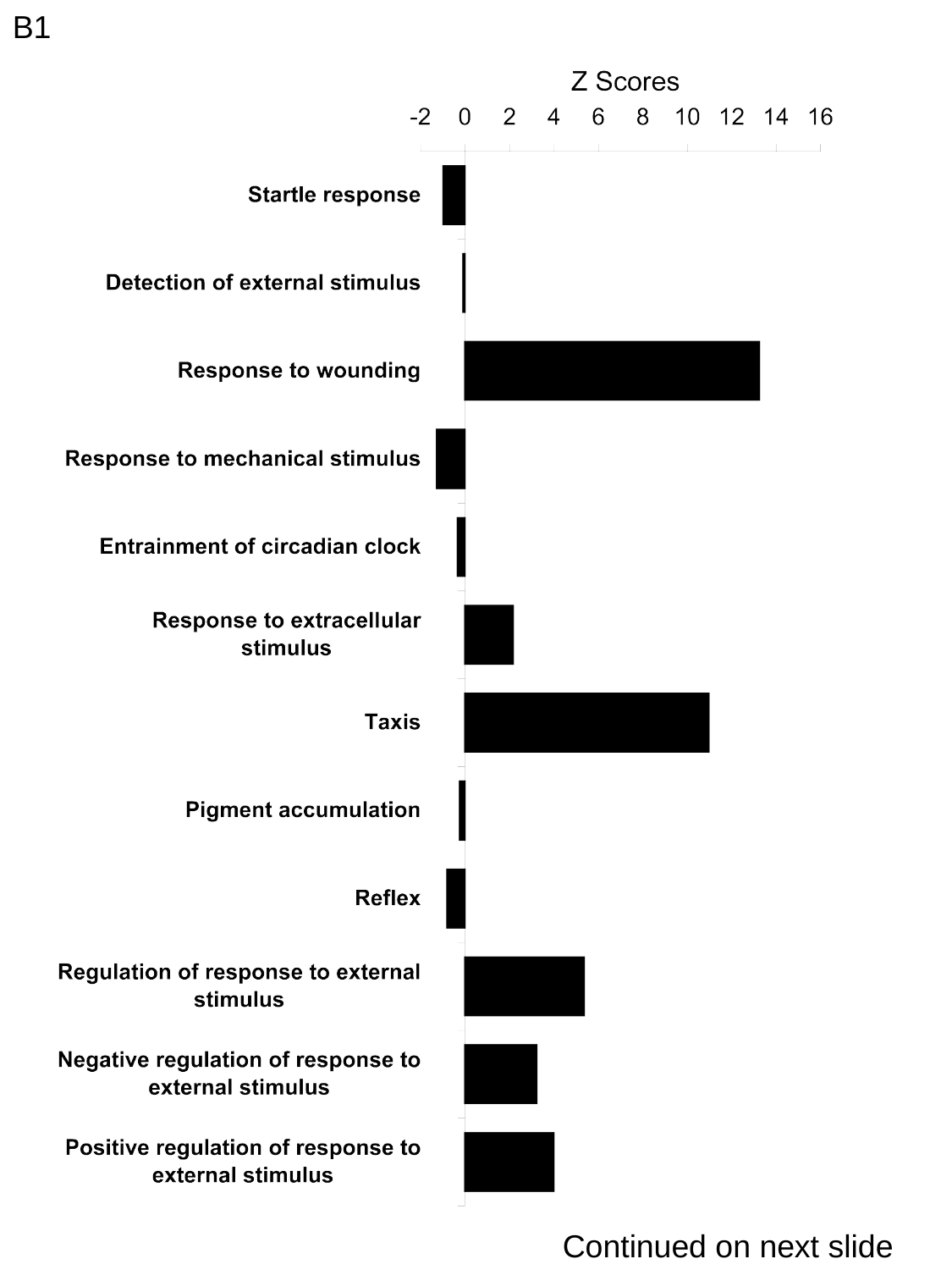

B1
Continued on next slide

## Slide 3
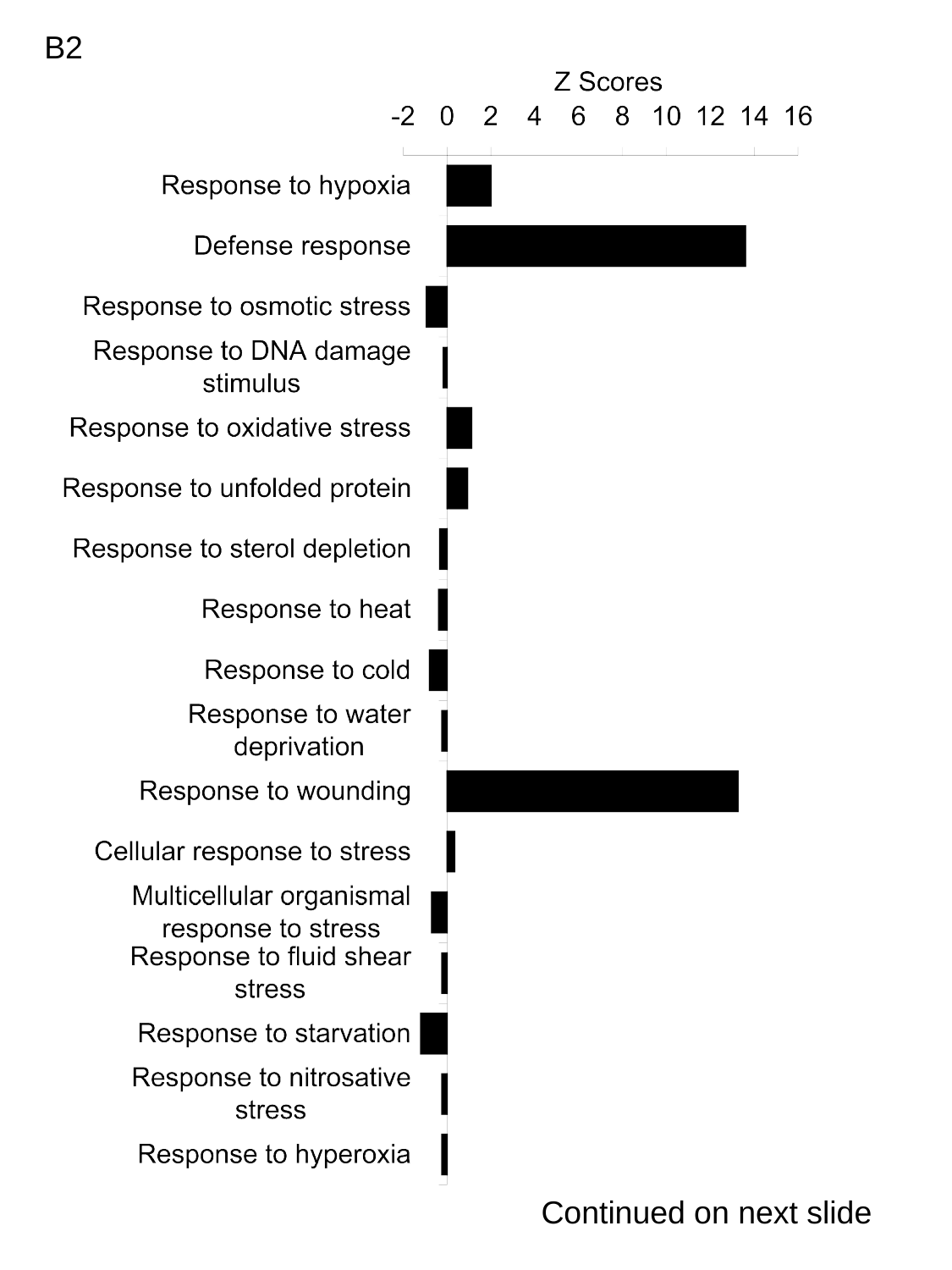

B2
Continued on next slide

## Slide 4
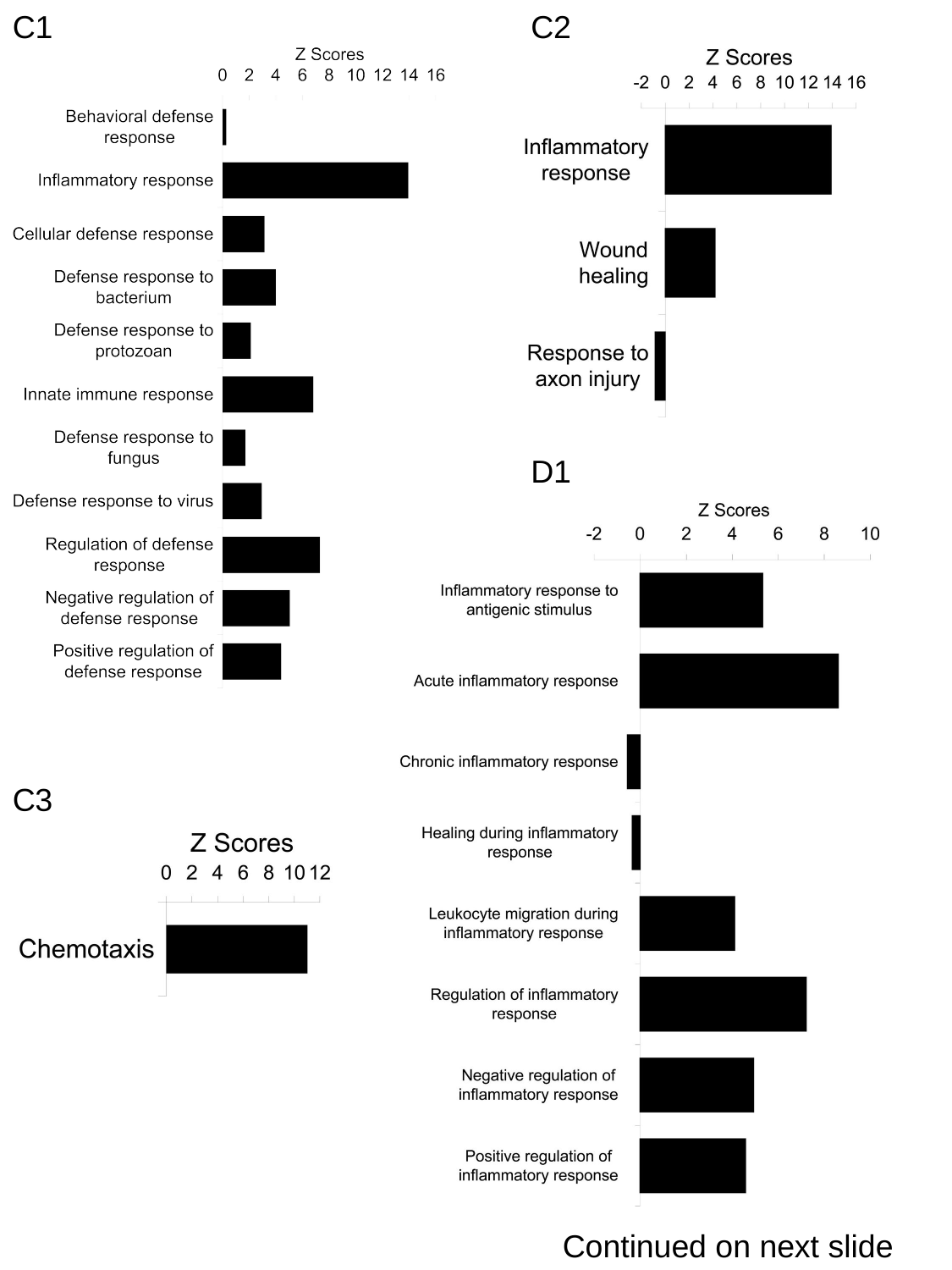

C1
C2
D1
C3
Continued on next slide

## Slide 5
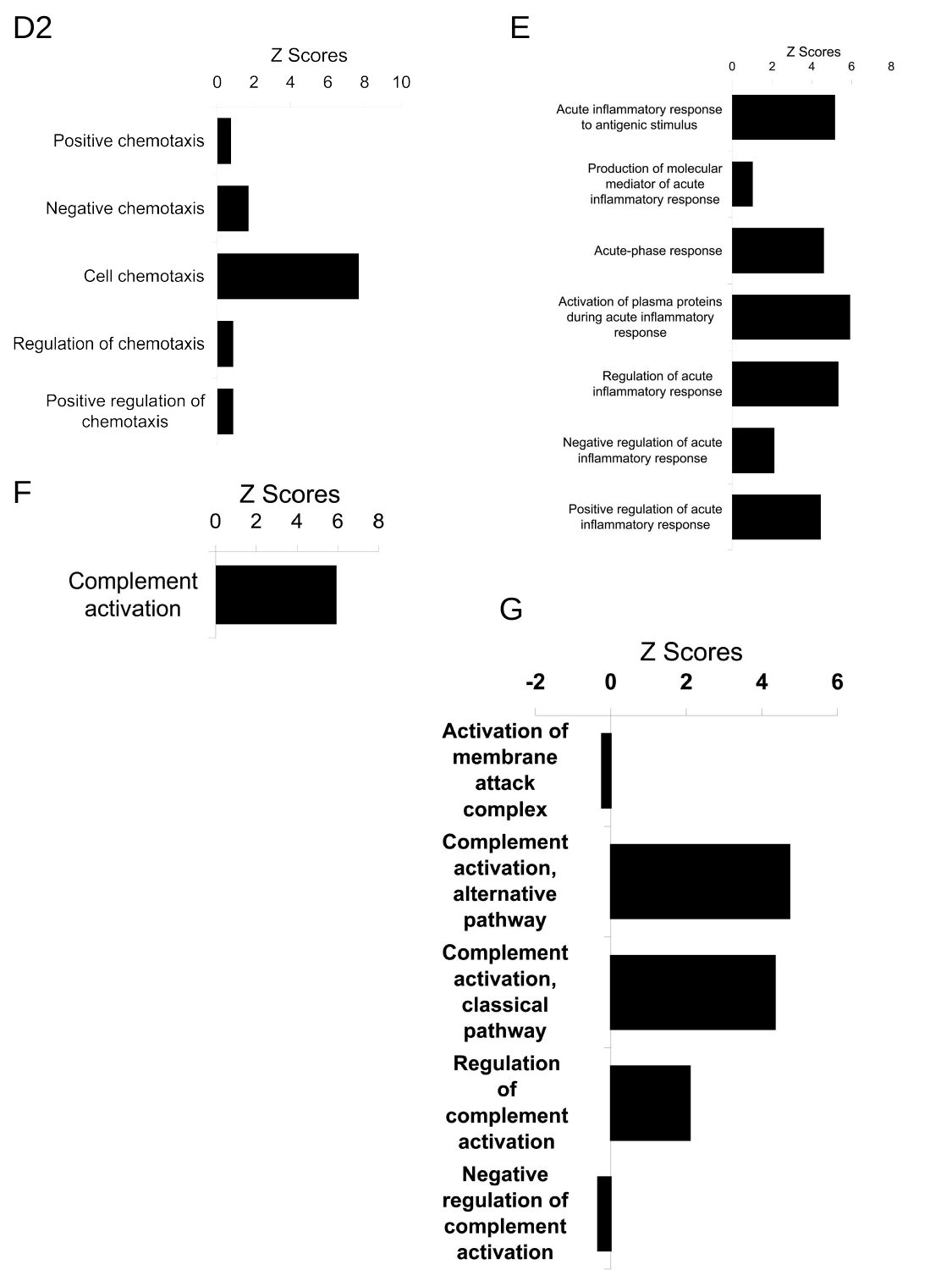

D2
E
F
G
